# Predicting Real-world Hypoglycemia Risk in American Adults With Type 1 or 2 Diabetes Mellitus Prescribed Insulin and/or Secretagogues: Protocol for a Prospective, 12-Wave Internet-Based Panel Survey With Email Support (the iNPHORM [Investigating Novel Predictions of Hypoglycemia Occurrence Using Real-world Models] Study)

**DOI:** 10.2196/33726

**Published:** 2022-02-11

**Authors:** Alexandria Ratzki-Leewing, Bridget L Ryan, Guangyong Zou, Susan Webster-Bogaert, Jason E Black, Kathryn Stirling, Kristina Timcevska, Nadia Khan, John D Buchenberger, Stewart B Harris

**Affiliations:** 1 Department of Epidemiology and Biostatistics Schulich School of Medicine and Dentistry Western University London, ON Canada; 2 Department of Family Medicine Schulich School of Medicine and Dentistry Western University London, ON Canada; 3 Robarts Research Institute Western University London, ON Canada; 4 Ipsos Healthcare New York, NY United States

**Keywords:** severe hypoglycemia, nonsevere hypoglycemia, type 1 diabetes mellitus, type 2 diabetes mellitus, real-world, risk model, risk prediction, hypoglycemia, symptom, diabetes, risk, model, protocol, survey, internet survey, adverse event, insulin, secretagogue

## Abstract

**Background:**

Hypoglycemia prognostic models contingent on prospective, self-reported survey data offer a powerful avenue for determining real-world event susceptibility and interventional targets.

**Objective:**

This protocol describes the design and implementation of the 1-year iNPHORM (Investigating Novel Predictions of Hypoglycemia Occurrence Using Real-world Models) study, which aims to measure real-world self-reported severe and nonsevere hypoglycemia incidence (daytime and nocturnal) in American adults with type 1 or 2 diabetes mellitus prescribed insulin and/or secretagogues, and develop and internally validate prognostic models for severe, nonsevere daytime, and nonsevere nocturnal hypoglycemia. As a secondary objective, iNPHORM aims to quantify the effects of different antihyperglycemics on hypoglycemia rates.

**Methods:**

iNPHORM is a prospective, 12-wave internet-based panel survey that was conducted across the United States. Americans (aged 18-90 years) with self-reported type 1 or 2 diabetes mellitus prescribed insulin and/or secretagogues were conveniently sampled via the web from a pre-existing, closed, probability-based internet panel (sample frame). A sample size of 521 baseline responders was calculated for this study. Prospective data on hypoglycemia and potential prognostic factors were self-assessed across 14 closed, fully automated questionnaires (screening, baseline, and 12 monthly follow-ups) that were piloted using semistructured interviews (n=3) before fielding; no face-to-face contact was required as part of the data collection. Participant responses will be analyzed using multivariable count regression and machine learning techniques to develop and internally validate prognostic models for 1-year severe and 30-day nonsevere daytime and nocturnal hypoglycemia. The causal effects of different antihyperglycemics on hypoglycemia rates will also be investigated.

**Results:**

Recruitment and data collection occurred between February 2020 and March 2021 (ethics approval was obtained on December 17, 2019). A total of 1694 participants completed the baseline questionnaire, of whom 1206 (71.19%) were followed up for 12 months. Most follow-up waves (10,470/14,472, 72.35%) were completed, translating to a participation rate of 179% relative to our target sample size. Over 70.98% (856/1206) completed wave 12. Analyses of sample characteristics, quality metrics, and hypoglycemia incidence and prognostication are currently underway with published results anticipated by fall 2022.

**Conclusions:**

iNPHORM is the first hypoglycemia prognostic study in the United States to leverage prospective, longitudinal self-reports. The results will contribute to improved real-world hypoglycemia risk estimation and potentially safer, more effective clinical diabetes management.

**Trial Registration:**

ClinicalTrials.gov NCT04219514; https://clinicaltrials.gov/ct2/show/NCT04219514

**International Registered Report Identifier (IRRID):**

DERR1-10.2196/33726

## Introduction

### Background

Although prognostic models can complement clinical decision-making and risk-tailored interventions [1-5], their performance depends heavily on the attributes of their underlying data sources [6]. The prognostic literature on diabetes-related hypoglycemia—a potentially lethal [7,8] and costly [9-11] side effect of insulin and/or secretagogues—has been dominated by analyses of pre-existing trial [12] or administrative databases [13]. However, these sources poorly represent high-risk diabetes populations [14-18], underestimate up to 95% of hypoglycemia events [14,19,20], and limit substantive evidence on potential predictors [21].

Prospective, web-based survey data, especially when collected anonymously [22], can reveal robust indications of hypoglycemia burden [23-26] routinely unmeasured or uncapturable by other research methods [20]. Such insight could help rectify extant evidence gaps, leading to more valid, real-world event prognostication [27] and, ultimately, targeted, cost-effective strategies that support hypoglycemia prevention in broad clinical contexts.

In 2020, our team launched iNPHORM (Investigating Novel Predictions of Hypoglycemia Occurrence Using Real-world Models)—the first prospective (1-year) survey of hypoglycemia risk in the American public with type 1 diabetes mellitus (T1DM) and type 2 diabetes mellitus (T2DM) prescribed insulin and/or secretagogues. The results of this study will culminate in real-world hypoglycemia prognostic models that are readily compatible with and complementary to routine practice. Here, we detail the design and implementation protocol of iNPHORM. The paper has been structured according to established guidelines [28,29] and the CHERRIES (Checklist for Reporting Results of Internet E-Surveys) guidelines [30].

### Objectives of the iNPHORM Study

#### Coprimary Objectives

The primary objectives are as follows:

1. To determine the real-world incidence of self-reported 1-year severe and 30-day nonsevere daytime and nocturnal hypoglycemia among American adults with T1DM or T2DM prescribed insulin and/or insulin secretagogues
2. To develop and internally validate real-world hypoglycemia risk prediction models for 1-year severe, 30-day nonsevere daytime, and 30-day nonsevere nocturnal hypoglycemia, which will be converted into a user-friendly, clinic-based tool

#### Secondary Objective

The secondary objective is to assess treatment-related causes of hypoglycemia among American adults with T1DM or T2DM prescribed insulin and/or insulin secretagogues.

## Methods

### Study Design and Setting

iNPHORM is an internet-based panel survey that was conducted across the United States. Repeated self-assessed measures were taken over 12 monthly interwave intervals via web-based questionnaires. Prospective longitudinality allowed us to (1) obtain data not reliably collected retrospectively or cross-sectionally (eg, variability in totals/averages or low-salience events), (2) assess within-person changes or stability masked by aggregate statistics, and (3) narrow the SE between measurements.

### Participants and Sample Size

Participants were recruited via the web from an established, closed, probability-based internet panel. The internet panel comprised 5 vendor samples of the United States public consenting to receive survey notifications by email (sample frame). Vendor partners used random probability sampling and, when necessary, validity checks, quotas, and multidimensional calibration. These approaches helped maintain fair and representative (geodemographic, attitudinal, and behavioral) sampling within communities [31]. The internet panel comprised >65,000 Americans with self-reported T1DM (N=10,000 approximately) and T2DM (N=58,000 approximately).

Internet panelists could enroll if they were (1) aged 18 to 90 years, (2) living in the United States (past year), (3) self-reporting a diagnosis of T1DM or T2DM [32], and (4) using insulin, secretagogues, or both insulin and secretagogues (past year). Individuals were ineligible if they were unable to read and understand English, possessed insufficient computer and internet literacy, or were participating in a concurrent trial. Those who were pregnant (at screening or in the prior year) and/or those with gestational diabetes were excluded, given their distinct pathogenesis and clinical management.

On the basis of recent conservative techniques [33,34], N≥521 respondents would be required to produce a 25-factor prognostic model for severe hypoglycemia (the rarest event type) with sufficient precision and minimal overfitting with ≤0.05 expected optimism [34,35]. Anticipating a degree of right censoring [35,36], we inflated our target sample to 1250 enrollees.

### Sampling, Recruitment, and Data Collection

**Figure 1:**
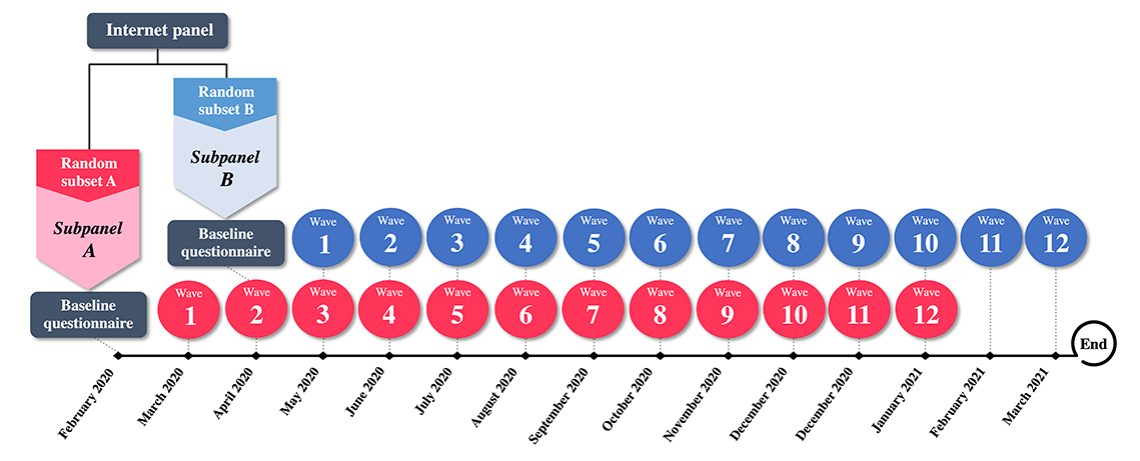
Schematic of participant sampling, recruitment, and data collection.

Figure 1 summarizes participant sampling, recruitment, and data collection.

A total of 2 subpanels (*A* and *B*) were recruited into the prospective, 12-wave iNPHORM study using convenience sampling. First, vendor partners emailed a generally worded study invitation to a randomly selected subset of the internet panel (subset A). Those interested were emailed a link to a screener. To enroll, eligible respondents were required to provide consent (see *Ethical Considerations* section), complete a baseline questionnaire (accessible by the emailed link), and register with iNPHORM using a confirmed, valid email address and unique username/password. Enrollees were hosted and monitored by Ipsos Interactive Services (IIS) [37], a global leader in diabetes insights and patient-centered, real-world survey conduct.

Links to the screener and baseline questionnaires remained active until we reached 1250 enrollees (ie, *subpanel A*). Participants in *subpanel A* who failed to complete the first wave follow-up questionnaire were withdrawn and systematically refreshed with new eligible recruits (ie, *subpanel B*). *Subpanel B* was sampled and enrolled in the same way as *subpanel A* but from a different, randomly selected subset (subset B) of the contemporaneous internet panel. Screener and baseline links remained active for approximately 2 weeks or until a 1:1 ratio of *subpanel B* to *subpanel A* wave 1 dropouts was achieved (whichever came first). Collectively, individuals in *subpanel A* who completed the first follow-up questionnaire and all those in *subpanel B* comprised the *iNPHORM longitudinal panel*.

Quota sampling ensured prespecified minimum parameters of the *iNPHORM longitudinal panel*. We required that ≥10% of participants report T1DM, ≥5% are aged ≥75 years, and ≥10% are female/male. Among T2DM respondents, we specified a ≥10% representation for insulin (without secretagogues), secretagogues (without insulin), and a combination of insulin and secretagogue users each.

We followed the *iNPHORM longitudinal panel* for 12 months. The calendar schedule between subpanels was identical; however, systematic refreshment caused follow-up waves to offset by 2 months (*subpanel A*: February 2020 to January 2021; *subpanel B*: April 2020 to March 2021). At each wave, IIS emailed participants an individualized link to a closed, fully automated questionnaire that involved no face-to-face contact. The link could only be accessed by the email recipient using their *iNPHORM longitudinal panel* username/password. Links were active for 7 days from distribution (activation window). The responses were synchronously stored on the IIS platform. Completed questionnaires could not be reaccessed or modified.

### Notifications, Precontacts, and Reminders

Personalized notifications, precontacts, and reminders were emailed automatically by IIS. Each notification contained the questionnaire link, the deadline for submission, and details on remuneration (see *Incentivization Scheme* section). Notifications also included the date of the participant’s last completed questionnaire, as well as their last reported use/type of antihyperglycemic(s) and glucose monitoring device(s).

To boost completion rates [38,39], a precontact alerting participants of an upcoming questionnaire was emailed 7 days before the notification. After the notification, individuals were sent 2 reminder emails on days 4 and 6 of the 7-day activation window. Reminders contained the same information as the corresponding notification emails.

### Incentivization Scheme

**Figure 2:**
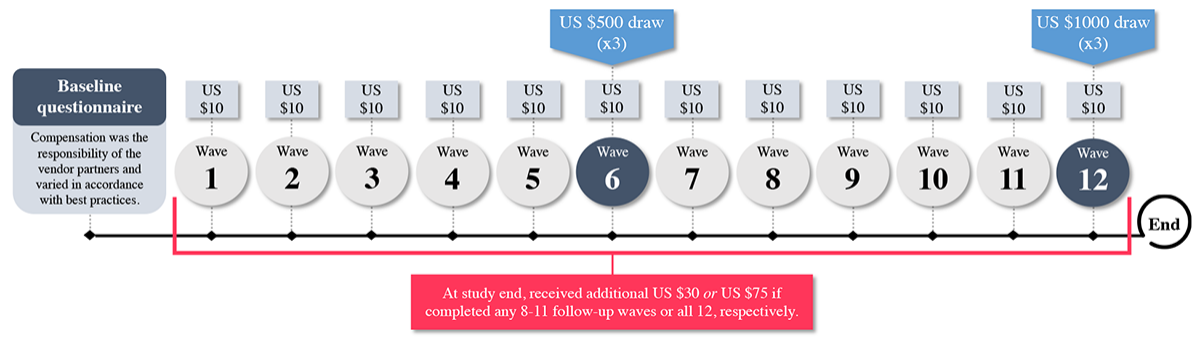
Incentivization scheme.

Figure 2 summarizes participant honoraria.

A thank you message and link to a US $10 e-gift card was emailed after each submitted follow-up. At the end of the study, participants received an additional e-gift card of US $30 if they completed any 8 to 11 waves or US $75 if they completed all 12 waves. Wave 6 and 12 responders were entered to win 1 of 3 randomly selected US $500 or US $1000 e-gift cards, respectively.

Incentive amounts balanced our desired response rates against ethical standards of reciprocity [40]. For internet-based surveys, monetary versus other inducements can decrease volunteer bias [35,36] and respondent refusals [41-43]. Lottery incentivization has been shown to act much like cash incentives with a value effect equal to the lottery prize divided by the sample size [44].

### Questionnaire Development Procedures

Western University scientists (AR-L, BLR, and SBH) developed questionnaires in consultation with the literature and pre-existing surveys. Questionnaires were designed in English for use on diverse internet-equipped devices (eg, computers, phones, and tablets). The content was crafted parsimoniously to lessen panel fatigue, conditioning, satisficing, social desirability bias, and demand characteristics [38]. Double-barreled questions, clinical jargon, and value-laden or complex/ambiguous language were avoided. We also ensured that the items were mutually exclusive, exhaustive, and specified an appropriate and consistent level of detail. Key questions were prioritized early; conversely, all sensitive items—justified and respectfully crafted (eg, income was categorized)—were interspersed to encourage respondent honesty [45]. We did not randomize/alternate items within or between questionnaires or participants. When applicable, items addressed the causal ordering of sequence, timing, and duration [46]. Recall intervals balanced the observation probability against the timing of questionnaire completion.

Established design principles were adopted to minimize burden and sustain engagement. Clearly worded preambles signaled topic changes and explained the importance of respondent honesty and vigilance [39,47]. To mitigate comprehension bias, concise instructions and definitions were provided in text and on mouseover [47]. In addition, efforts were taken to enhance accessible visual appeal, navigation, and user convenience. Adaptive questioning streamlined transitions between items and decreased the complexity and length (ie, number of screens) of the web interface questionnaires. For ease of completion, straightforward response options (via radio buttons, checkboxes, drop-down lists, and open-text fields) were presented, and only 1 item appeared per screen. Questionnaires could be accessed, delayed, and/or paused ad libitum up until submission or the activation window closed (whichever came first). Percentage-based progress bars on each screen supplied visual feedback on completion.

Quality assurance methods were applied to reinforce data integrity. *Calibration* questions [48] were incorporated in the screener to detect straight lining, verify item comprehension, and avert nonsensical free text [49]; unsatisfactory answers precluded participant enrollment. In-built logic checks supported data accuracy [49]. For example, questions were prespecified with single- or multi-responses, and *not applicable, prefer not to say*, and *I don’t know* were delimited as exclusive options. Missing responses were immediately flagged. To bypass a question, individuals had to type “OPT OUT” in a pop-up response box, helping discriminate intentional nonresponse from inadvertent omissions/straight lining. At the start of every questionnaire, respondents were reminded to retrieve any documents/materials that could facilitate response accuracy (eg, medication lists/containers and glucose monitoring logs/graphs).

During follow-up, IIS monitored bugs, downtimes, and other unexpected events that could have affected the study design. At any point, participants could email IIS Technical Support (email address was included in all iNPHORM communications).

### Pretesting and Piloting

iNPHORM researchers and colleagues performed extensive pretesting of detailed mock-up and programmed study materials to redress issues of content, display, adaptive questioning, and implementation. Before their dissemination, programmed questionnaires, notifications, and reminders were piloted via in-depth semistructured interviews with 3 participants who were screened and sampled purposively from a subset different than subsets A and B of the internet panel. Of the 3 participants, 1 (33%) participant had T1DM; the other 2 (67%) had T2DM (1, 50%, was prescribed secretagogues without insulin, and 1, 50%, a combination of insulin and secretagogues). A trained IIS moderator (JDB) interviewed participants simultaneously by phone and a computer-assisted personal interview platform using an interview guide developed by the Western University research team.

Qualitative feedback was collected on content, formatting, flow, usability, and technical functionality. Pilot data were also gathered on sample variability, item response rate, and time to completion. Behaviors signaling design issues were documented (eg, instances where the respondent hesitated or requested to change an answer) [38]. Interviews took 60 to 90 minutes. The study materials were emended based on respondents’ feedback. Pilot participants were remunerated US $300 (e-gift card); they were not permitted to enroll in the panel survey.

Once finalized and in field, no changes were made to questionnaires except for the addition of a COVID-19 subquestionnaire (see the *COVID-19 Subquestionnaire* section). Dynamic components were obviated to preserve study replicability.

### Prognostic Factors Related to Hypoglycemia and COVID-19

#### Overview

Across the screener, baseline, and follow-up questionnaires, web-based self-assessed data were collected on a broad scope of hypoglycemia-related anthropometric, demographic, situational or environmental, lifestyle (Multimedia Appendix 1), and clinical (Multimedia Appendix 2 [50-53]) prognostic factors. Follow-up questionnaires also contained items related to COVID-19 (Multimedia Appendix 3; see *Definitions and Measures of Hypoglycemia* section for methods of hypoglycemia-specific data capture).

#### Screener

The pilot screener took an average of 9.6 (SD 4.73; minimum 6 and maximum 15) minutes to complete. Data were collected on age, sex assigned at birth, self-identified gender, residence, concurrent trial involvement, diabetes type, pregnancy status, and insulin and/or secretagogue use (eg, administration mode [when applicable], dose, and duration). Response options for medication type were arranged by class, save second-generation basal insulin analogs, which were listed by brand (Toujeo SoloSTAR, Toujeo Max SoloStar, Tresiba FlexTouch U-100, and Tresiba FlexTouch U-200). Screener data were retained for all consenting individuals.

#### Baseline Questionnaire

On average, pilot respondents completed the baseline questionnaire in 47.3 (SD 13.65; minimum 38 and maximum 63) minutes. Information was elicited on anthropometric, demographic, situational or environmental, and lifestyle factors (eg, levels of aerobic/anaerobic activity and cigarette, alcohol, and recreational drug use). Numerous clinical data were also collected on diabetes duration, diabetes self-management behaviors, diabetes complications (eg, chronic kidney disease), general health status (eg, chronic multi-morbidities and use of dialysis), and health-related quality of life.

To simplify future population-based comparisons and statistical weighting, we devised items with reference to existing population-based surveys by the US Census Bureau (2020) [54] and the Centers for Disease Control and Prevention (ie, National Health and Nutrition Examination [2019-2020] [55], Behavioral Risk Factor Surveillance System [2020] [56], and National Health Interview Survey [2020] [57]). We also embedded several validated questionnaires (eg, Veterans RAND-12 [50,53], Self-Rated Health [51], and Brief Health Literacy Screening Tool [52]).

#### Follow-up Questionnaires

Follow-ups (except wave 6 see *Definitions and Measures of Hypoglycemia* section) were on average piloted in 10.8 (SD 5.30; minimum 7 and maximum 14.5) minutes. Items assessed mutable clinical variables (eg, medication regimen, hemoglobin A1c, and continuous/flash glucose monitoring). Employment status, household income, and health insurance were re-evaluated at waves 4, 8, and 12.

#### COVID-19 Subquestionnaire

Pandemic-related items were added after study commencement in response to the escalating severity of the COVID-19 pandemic. Beginning with *subpanel A* wave 2 (April 21 to April 28, 2020), each follow-up contained a 25-item COVID-19 subquestionnaire that assessed self-reported infection status (per Centers for Disease Control and Prevention’s community case definitions [April 2020]; [58]) and the impact of the pandemic situation on socioeconomic, clinical, and psychosocial aspects of diabetes management [59].

### Definitions and Measures of Hypoglycemia

At baseline and at each follow-up (Multimedia Appendix 4 [60-63]), web-based self-assessed data were collected on severe and nonsevere daytime and nocturnal hypoglycemia; definitions consistent with the 2019 American Diabetes Association Standards of Medical Care in Diabetes [64] were provided in all questionnaires (Textbox 1).

In line with past research [60,65-67], we specified interwaves of ≤1 year for severe and ≤30 days for nonsevere hypoglycemia. At baseline, participants were asked to report on their severe daytime/nocturnal hypoglycemia in the past year and nonsevere daytime/nocturnal hypoglycemia in the past 30 days. To prevent overlapping recall intervals during follow-up, data on nonsevere daytime and nocturnal hypoglycemia were captured *within the past 30 days* (if the last scheduled questionnaire was not completed) or *since the last time an iNPHORM survey was completed* (if the last scheduled questionnaire *was* completed). Given its relative infrequency and saliency, severe daytime and nocturnal hypoglycemia data were captured *since the last time an iNPHORM survey was completed*.

Besides hypoglycemia frequency, closed- and open-ended items assessed event detection methods (eg, symptoms and/or blood glucose), symptom severity (eg, unconsciousness), causes (eg, excess insulin and/or secretagogue use, insufficient carbohydrate intake, and excess physical activity), treatments, hypoglycemia-specific self-management behaviors/social support, and experiences with continuous/flash glucose monitoring. We also investigated the type of assistance required for severe hypoglycemia recovery (eg, treatment by family/friend and health care use). Each month, modified Clarke [61] and Gold [62] scores evaluated impaired hypoglycemia awareness. At wave 6, we administered the Hypoglycemia Fear Survey II [63] and the InHypo-DM Person with Diabetes Questionnaire [60].

**iNPHORM (Investigating Novel Predictions of Hypoglycemia Occurrence Using Real-world Models) hypoglycemia definitions provided to participants by severity and timing.**

**Severe**

- “When you are *physically unable* to treat your hypoglycemia by yourself, it is considered a *Severe Hypoglycemia* event. You may be severely disorientated, unable to swallow, or unconscious. As a result, you are likely to need the help of another person to recover. This person may need to administer glucagon or a glucose injection to treat your severe hypoglycemia event. Emergency medical services may be called, and hospitalization may be required. Severe events can arise when your low blood glucose is left untreated and continues to drop. The early signs and symptoms of severe hypoglycemia typically include blurred vision, difficulty concentrating, confused thinking, slurred speech, numbness, and/or drowsiness. If your blood glucose stays low for too long, it can result in seizures, comas, and in rare cases, death. Consequently, severe hypoglycemia is a medical emergency.”

**Mild/moderate (also known as nonsevere)**

- “When you are *physically able* to treat your hypoglycemia by yourself, it is considered a *Mild/Moderate Hypoglycemia* event. Treatment can include taking a glucose or sucrose tablet, drinking a glass of juice, or eating some food. Mild/moderate hypoglycemia events can be identified by symptoms such as shakiness, sweatiness or chills, irritability, feeling nervous or anxious, hunger, weakness, mild confusion, forgetfulness, fast heartbeat, feeling dizzy, and color draining from the skin. Mild/moderate hypoglycemia events can be identified from these symptoms or by a measured blood glucose level taken from a self-monitoring blood glucose (SMBG) meter or continuous/real-time glucose monitoring (CGM) device. You are still conscious and able to swallow.”

**Daytime**

- “*Daytime events* (mild/moderate or severe) occur while you are awake.”

**Nocturnal**

- “*Nocturnal events* (mild/moderate or severe) occur while you are sleeping or attempting to sleep. In addition to the symptoms described above, nocturnal hypoglycemia can be marked by symptoms such as vivid dreams/nightmares, restless sleep, morning headaches, night sweats, tiredness, irritability/confusion upon waking, convulsions, and talking/shouting while sleeping.”

### Ethical Considerations

iNPHORM was funded by an investigator-initiated grant from Sanofi Global (contract executed with Sanofi Canada, April 11, 2019). Before recruitment, we obtained ethics approval from the Western University health sciences research ethics board (December 17, 2019) and registered the study with ClinicalTrials.gov (NCT04219514; January 7, 2020). The COVID-19 subquestionnaire was approved as an ethics amendment before fielding.

A letter of information was emailed to all eligible respondents (Multimedia Appendices 5 and 6). The letter named Western University as the responsible academic institution and Sanofi Canada as the funding agency. It also outlined the study’s purpose, nature and expectations of participation (eg, content of surveys, time commitment, follow-up frequency, and incentivization), risks and benefits, participant rights (eg, refusals/withdrawals), and confidentiality/privacy measures (eg, data storage, retention, sharing, and reporting). Contacts were provided for IIS, faculty coprincipal investigator (SBH), Western University research team, and the Office of Human Research Ethics at Western University. Conflicts of interest for SBH have been declared. Consent was obtained via the web. Individuals were advised to read the letter of information before clicking on *I agree to participate* or *I do not agree to participate*.

Participation was voluntary. Enrollees could withdraw at any time by informing the IIS interviewer (pilot participants only), clicking an unsubscribe button provided in each email, or by emailing IIS directly. Privacy breaches and technical problems were monitored by IIS. Personally identifiable data (eg, phone numbers [pilot participants only], email addresses, and full birthdates) were encrypted automatically by the IIS platform and kept confidential from IIS and research personnel. IIS transferred deidentified data files to the Western University research team using a secure file transfer protocol on a password-protected network drive. All deidentified data will be stored for 7 years on a password-protected network drive at the Department of Family Medicine at Western University and on encrypted password-protected external drives; storage devices will be erased after this time. The iNPHORM assessments and data are owned by Western University.

Complying with US Food and Drug Administration postmarket safety reporting regulations [68], we emailed Sanofi United States and Novo Nordisk United States monthly pharmacovigilance reports of severe adverse events among Toujeo and Tresiba users, respectively. The reports were anonymized.

### Planned Statistical Analyses

#### Overview

Unique IDs, randomly assigned by IIS at the study outset, were used to tether the participants’ data across waves. Closed-ended responses were directly precoded, and a data dictionary and map have been developed. Repair rules addressing impossible, implausible, and discordant values will be documented in iNPHORM’s metadata (eg, erroneous responses will be classified as missing or cross-checked against valid responses). Both the raw and repaired data sets will be retained.

#### Describing the iNPHORM Sample

##### Recruitment and Completion Rate

The recruitment rate will be calculated as the ratio of consenting individuals to enrollees. The average total completion rates for the *iNPHORM longitudinal panel* will be computed as the ratio of the observed number of completed waves to the maximum expected number (12 waves per participant). To evaluate the success of our completion rate against our predetermined sample size (N=521; *Sample Size* section), the observed number of waves for which severe hypoglycemia information was available will be compared against the maximum expected number of completed follow-ups.

##### Completeness Rate

All data were stored in real time for analysis, even if the questionnaire was incomplete (eg, prematurely terminated). The completeness rate will be assessed after data cleaning and repair. Missing values will be coded as unit, block, item (because of skip logic), or residual (because of *not applicable*/*prefer not to say*/*I don’t know* or opt out) nonresponses. Missing data will be handled using multiple imputation by chained equations [69].

##### Participant Characteristics

Categorical variables will be summarized as frequencies and percentages, and continuous variables as means and SDs (parametric) or medians and IQRs (nonparametric).

#### Hypoglycemia Incidence (Coprimary Objective 1)

Crude severe and nonsevere daytime and nocturnal hypoglycemia incidence proportions and densities with 95% CIs for overdispersed count data will be reported overall and by diabetes type, medication regimen, mode of detection (symptoms and/or blood glucose), symptom severity (unconsciousness), and health care use. Incidence density calculations will account for observation durations as an offset for zero-risk and/or unobserved periods.

#### Prognostic Model Construction (Coprimary Objective 2)

##### Overview

The following procedures comply with current guidelines [70,71] and the Transparent Reporting of a Multivariable Prediction Model for Individual Prognosis or Diagnosis statement [72,73]. Analyses will be performed on baseline respondents who submitted ≥1 follow-up questionnaire. To pre-empt statistical power loss and selection bias, all baseline and follow-up data on this cohort will be examined [74]. Iterative proportional fitting (raking) [38] to correct for nonresponse and unequal selection probability will be investigated.

##### Model Development

Prognostic models will be developed for severe, nonsevere daytime, and nonsevere nocturnal hypoglycemia. Daytime and nocturnal severe events will be combined, given their nonspecific relevance and to ensure sufficient precision. Severe hypoglycemia will be modeled over 1 year using the Andersen-Gill Cox proportional hazards regression for recurrent events [34]. Nonsevere daytime and nocturnal hypoglycemia will be modeled over 30 days using negative binomial regression. Observation duration will be included as an offset variable, and generalized estimating equations will account for within-person dependence.

Candidate prognostic factors will be selected a priori based on biological plausibility, previous literature, data quality, measurement reliability, and multicollinearity. Intrinsic, extrinsic, nonmodifiable, and modifiable predictors (including frequency of previous severe and nonsevere hypoglycemia) will be considered. To minimize overfitting [75,76] and improve parsimony, model parameters will be estimated using machine learning penalized regression with Lasso (least absolute shrinkage and selection operator) [77]. Regression splines and fractional polynomials will assess the potential for nonlinearity and nonmonotonicity [78]. Interaction and subgroup analyses will be performed where suggested by external evidence [2]; sensitivity analyses will test the robustness of the findings. Informative censoring will be explored using inverse probability of censoring weighted estimation [79,80].

##### Internal Validation

Bootstrapping will be used to determine the optimism-corrected performance of each final model [74,77,81]. Discrimination will be evaluated using receiver operating characteristic curves and c-statistics [82]. Calibration will be assessed visually (eg, via graphical plots) and quantified using the calibration slope, the Hosmer-Lemeshow goodness-of-fit test, and the Grønnesby and Borgan test for survival data [83-85].

##### Pragmatic Tool Creation

Models will be converted into a user-friendly, clinic-based tool to complement real-world practice. Back-end computations of patients’ prognostic factors will provide point-of-care assessments for 1-year severe and/or 30-day nonsevere daytime/nocturnal hypoglycemia. To aid interpretation, risk estimates will also be categorized (eg, low, moderate, high, and very high).

The tool will be streamlined for easy integration in clinicians’ existing electronic medical records (EMRs) and compatible with prepopulated EMRs and manually inputted data. A standalone internet application and paper-based nomogram will be developed for when EMR integration is not possible. Real-time imputation will be explored [86].

#### Treatment-Related Causes of Hypoglycemia (Secondary Objective)

Differential effects of antihyperglycemic regimens on hypoglycemia rates will be tested using causal analytic techniques (eg, directed acyclic graphs, parallel and serial mediation, and time-dependent confounding). The results may help in identifying new and useful associations that can improve model performance or otherwise real-world event detection and management [87].

## Results

### Overview

**Figure 3:**
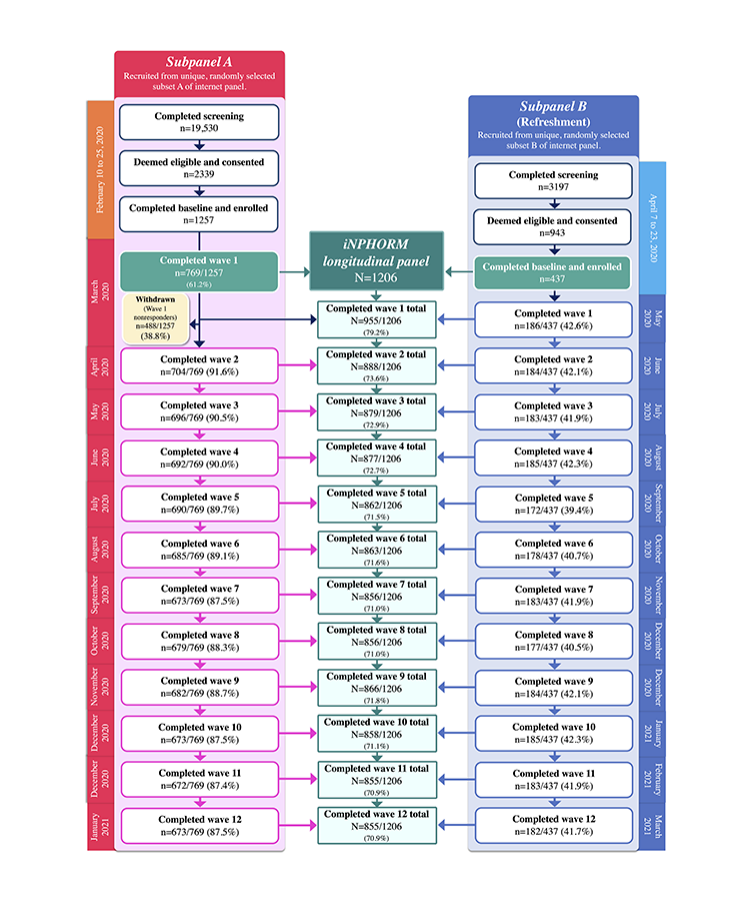
Recruitment and completion rates. iNPHORM: Investigating Novel Predictions of Hypoglycemia Occurrence Using Real-world Models.

iNPHORM commenced in February 2020 and concluded in March 2021. No bugs, downtimes, privacy breaches, or other unexpected events were reported/detected. Herein, we present the recruitment and completion rates (Figure 3). Analyses of participant characteristics and hypoglycemia incidence and prognostication are currently underway, with published results anticipated by fall 2022. Future studies will investigate the distributions of participant discontinuance [35] and systematically report on quality metrics, including missing values and data cleaning statistics, follow-up completeness [88], degree of coverage/sampling bias, and process outcomes (eg, average time-to-completion).

### Recruitment Rate

From February 10 to February 25, 2020, 2339 individuals consented to participate in iNPHORM; of these individuals, 1257 (53.74%) completed all actions to enroll (ie, *subpanel A*). Individuals in *subpanel A* who failed to complete wave 1 were withdrawn (488/1257, 38.82%) and systematically refreshed with *subpanel B*. From April 7 to April 23, 3197 individuals consented, of whom 437 (13.67%) were enrolled. Thus, as of April 2020, 1206 participants comprised the *iNPHORM longitudinal panel*.

### Completion Rate

The average total completion rate across the *iNPHORM longitudinal panel* was 72.4% (Multimedia Appendix 7). Given our use of systematic refreshment, *subpanel A* exhibited a higher completion rate than *subpanel B* (89.8% vs 41.6%, respectively). Dropout was highest at wave 1, with completion rates stabilizing thereafter. Across respondents, 71.89% (867/1206) completed ≥8 follow-ups, with 55.22% (666/1206) completing all 12 (Table 1). We observed minimal loss to follow-up (ie, individuals who discontinued participation until the end of the study). Most (855/1206, 70.9%) completed wave 12 (Table 2). Compared with our target sample size (N=521), we calculated a completion rate of 179% (Multimedia Appendix 8).

**Table 1:** Number of questionnaires completed overall and by diabetes type (N=1206).

Number of questionnaires completed^a^	Respondents, n (%)		
	Total	T1DM^b^ (n=194)	T2DM^c^ (n=1012)
Baseline only^d^	193 (16)	29 (14.9)	164 (16.2)
1-7	146 (12.1)	20 (10.2)	126 (12.5)
8-11	201 (16.7)	35 (18.2)	166 (16.4)
All 12	666 (55.2)	110 (56.7)	556 (54.9)

^a^Questionnaires completed could be nonconsecutive.

^b^T1DM: type 1 diabetes mellitus.

^c^T2DM: type 2 diabetes mellitus.

^d^Only *subpanel B* respondents; *subpanel A* respondents were removed upon wave 1 noncompletion.

**Table 2:** Number of respondents lost to follow-up after each wave overall and by diabetes type (N=1206).

Wave^a^	Respondents lost to follow-up after each wave, n (%)		
	Total	T1DM^b^ (n=194)	T2DM^c^ (n=1012)
Baseline^d^	193 (16)	29 (14.9)	164 (16.2)
Wave 1	33 (2.7)	8 (4.1)	25 (2.5)
Wave 2	17 (1.4)	2 (1)	15 (1.5)
Wave 3	10 (0.8)	1 (0.5)	9 (0.9)
Wave 4	14 (1.2)	0 (0)	14 (1.4)
Wave 5	7 (0.6)	0 (0)	7 (0.7)
Wave 6	5 (0.4)	3 (1.6)	2 (0.2)
Wave 7	8 (0.7)	0 (0)	8 (0.8)
Wave 8	6 (0.5)	1 (0.5)	5 (0.5)
Wave 9	8 (0.7)	1 (0.5)	7 (0.7)
Wave 10	12 (1)	0 (0)	12 (1.2)
Wave 11	38 (3.2)	9 (4.6)	29 (2.9)
Wave 12^e^	855 (70.9)	140 (72.2)	715 (70.7)

^a^Last wave responded to; after this wave, the respondent was considered to be lost to follow-up.

^b^T1DM: type 1 diabetes mellitus.

^c^T2DM: type 2 diabetes mellitus.

^d^Only *subpanel B* respondents; *subpanel A* respondents were removed upon wave 1 noncompletion.

^e^No data were collected past wave 12.

## Discussion

### Principal Findings

The real-world iNPHORM study is the first primary research investigation focused on quantifying and predicting prospective self-reported hypoglycemia in the United States. A general cohort of adult Americans with self-reported insulin- and/or secretagogue-treated T1DM or T2DM was recruited between February and April 2020 and followed for 1 year. The sample size was achieved using a 1-time systematic refreshment and quota sampling. The use of an established probability-based internet panel, push factors (precontacts, reminders, and incentives), and easy-to-complete questionnaires shored up high participation rates. Sample characteristics, quality metrics, and hypoglycemia incidence and prognostication will be published by fall 2022.

### Study Strengths

Poor generalizability has been an ongoing problem in prognostic hypoglycemia research [89]. To promote real-word representativeness and population inferencing, iNPHORM participants were recruited from random subsets of a well-established, probability-based internet panel. Community-based adults across a wide age range with either T1DM or T2DM, irrespective of past hypoglycemia, were eligible to enroll, as were people prescribed secretagogues, an often underappreciated cause of events [90]. Backstopped by quota sampling, our use of broad eligibility criteria stands in juxtaposition to most prognostic models [91], especially those based on pre-existing trial data, which focus on inpatient [18-21] or younger, healthier (eg, no severe hypoglycemia history or impaired awareness) [14,17] populations.

Data were collected over 12 one-month intervals, balancing the probability of observing events against participants’ abilities to recall them accurately. Frequent and long-term data capture enabled us to obtain maximally valid self-reported information on not only hypoglycemia occurrence but also a range of important, preselected factors commonly unavailable in secondary sources [92]. The longitudinal, prospective nature of our study contrasts the typically short, retrospective follow-ups of other prediction models (mode duration 24 hours-3 months) [12,93-96]. Buttressed by a sufficiently large sample size and completion rate >70%, iNPHORM will facilitate assessments of time-varying predictors, lagged dependent variables, and low-salience events (eg, nonsevere hypoglycemia) with minimal false negatives, extrapolation bias, and statistical power loss [97].

Our self-report study yields pertinent insights into the routinely uncaptured burden of hypoglycemia. Past prognostic hypoglycemia research has relied heavily on administrative, insurance-based claims records; however, these sources poorly represent events occurring outside the health care system. Recent evidence suggests that only 5% of severe events require hospitalization, and as many as 50% are treated at home by family/friends [19,20]. Moreover, nonsevere hypoglycemia, by definition *self-treated*, [98] is scarcely, if ever, documented. Patient nondisclosure and provider underrecognition further constrain the real-world applicability of epidemiological data gleaned from clinical encounters. Studies indicate that 65% and 85% of people with diabetes deliberately underreport their severe [99] and nonsevere [100] events, respectively, whereas 57% are seldom asked about hypoglycemia by their providers [99]. Not surprisingly, anonymous versus onymous hypoglycemia reporting has been associated with 2- to 3-fold higher rates [22].

iNPHORM builds on the methodological and economic advantages of real-time, web-based self-report to acquire instantaneous and representative [25,26] data within large samples [101]. Indeed, web-based questionnaires have been lauded for democratizing and potentiating self-report research. Currently, >90% of Americans use the internet [102]. iNPHORM data were collected via user-friendly, self-administered questionnaires completable on diverse internet-equipped devices at the participants’ convenience. Very little personal information was requested, and participants were made aware in the letter of information that their data would be deidentified before analysis. By forgoing dependence on health care codes and records, we could obtain real-world, granular information on severe (regardless of health care use) and nonsevere hypoglycemia—events rarely reported in the literature, despite their clinical significance.

### Limitations and Strategies to Mitigate Them

Certain limitations and safeguards warrant elaboration. Notwithstanding efforts to promote generalizability, selection biases could have arisen because of the nonrepresentativeness of the internet panel demography and/or of respondents/responses [36,103,104]. This concern affects correlative estimates less; however, it could distort the validity of summary statistics [105]. For this reason, post hoc statistical weighting will be explored [105]. Biases resulting from English language restriction, lack of technological literacy, being limited to no internet access, and survivorship cannot be discounted. Furthermore, although volunteer bias will be assessed during follow-up, baseline self-selection is not calculable (it was unethical to retain data on otherwise eligible invited panelists who did not complete the screener).

Another related limitation is the risk of attrition bias. To mitigate loss to follow-up, ostensibly unmotivated respondents in *subpanel A* were identified and removed at wave 1 via logic testing and noncompletion. One-time systematic refreshment, especially during the first interwave when attrition is highest, has been shown to reduce panel stagnation while improving study feasibility and analytic validity [38]. To prevent further biases, *subpanel B* was recruited from a contemporaneous subgroup of the same frame population as *subpanel A*. Push factors were used to sustain participation [35]. Remuneration coincided with the widely recognized Tailored Design Method by Dillman [106]. Cash amounts were vetted and approved by the Western University health sciences research ethics board before study commencement and outlined in the letter of information. Token incentives were strategized to facilitate revenue-neutral participation (eg, reasonably compensate individuals for their time and help overcome access barriers), reducing volunteer bias [35,36] and respondent dropout [41-43].

Although web-based (vs postal or telephone) surveys have been shown to promote item completeness and accuracy [23,24], they are not immune to recall bias. Research indicates that 90% [63] of patients correctly recall past-year severe hypoglycemia; however, past-month nonsevere hypoglycemia recall ranges from 48% to 75% [67]. To reduce differential misclassification bias, standardized, accessibly worded instructions and definitions were provided in each questionnaire. Furthermore, sensitive items were carefully crafted and positioned to encourage respondent honesty [45]. Technical constraints on the IIS platform precluded participants from reviewing or changing the submitted items. In addition, as mechanisms for deterring multiple participant identities, individuals could not reaccess/resubmit questionnaires, and authentication by email plus log-in was required. To foster confident and accurate responses, we provided individuals as much time as needed to reflect on items and/or review personal clinical documentation/materials. Each notification also contained information on the participants’ last completed questionnaire.

Before fielding, the assessments underwent pretesting and piloting to promote content usability and accuracy. A total of 3 individuals participated in the pilot process; this sample size aligned with established best practices at IIS while permitting parsimonious representativity and feasibility. Nevertheless, a larger pilot sample size may have yielded further meaningful feedback. Finally, despite the proven validity/reliability and/or widespread use of many iNPHORM items, no validated self-reported hypoglycemia measure exists yet. To attenuate instrumentation effects in our study [107], hypoglycemia definitions and classifications followed the 2019 American Diabetes Association standards [64], and recall periods echoed peer-reviewed conventions [60,65-67]. Frequent and recurrent hypoglycemia-related information was amassed across extensive, detailed, and standardized items formulated to promote scientific replicability and future outgrowth. The validity of iNPHORM is further fortified by high completion rates [108] and numerous design principles and quality assurance methods that reinforce data accuracy and integrity.

### Conclusions

iNPHORM promises important forward strides in real-world hypoglycemia detection and prevention. This protocol highlights the powerful application of an internet-based panel survey to assess long-term hypoglycemia risk in a large, community-based cohort of adult Americans with insulin- and/or secretagogue-treated T1DM and T2DM. To date, descriptive and prognostic hypoglycemia estimates have stemmed mainly from cross-sectional and short-term retrospective analyses of pre-existing databases subject to untenable bias. Pairing the importance of longitudinal, prospective self-reported hypoglycemia data with the advantages of web-based survey modes, iNPHORM aims to clarify putative epidemiological understandings and reveal opportune insights into point-of-care decision-making, research priorities, and effective interventional precision [109-111].

## Appendix

Multimedia Appendix 1 Anthropometric, demographic, situational or environmental, and lifestyle variables.

Multimedia Appendix 2 Clinical variables.

Multimedia Appendix 3 COVID-19–related variables.

Multimedia Appendix 4 Hypoglycemia-related variables.

Multimedia Appendix 5 Letter of information and consent emailed to prospective participants of the iNPHORM (Investigating Novel Predictions of Hypoglycemia Occurrence Using Real-world Models) pilot study.

Multimedia Appendix 6 Letter of information and consent emailed to prospective participants of the iNPHORM (Investigating Novel Predictions of Hypoglycemia Occurrence Using Real-world Models) longitudinal study.

Multimedia Appendix 7 Calculation of average total completion rate.

Multimedia Appendix 8 Calculation of average total completion rate against estimated required sample size (N=521).

## Abbreviations

EMR: electronic medical record
IIS: Ipsos Interactive Services
iNPHORM: Investigating Novel Predictions of Hypoglycemia Occurrence Using Real-world Models
Lasso: least absolute shrinkage and selection operator
T1DM: type 1 diabetes mellitus
T2DM: type 2 diabetes mellitus
