# Supplementary material for: Predicting Real-world Hypoglycemia Risk in American Adults With Type 1 or 2 Diabetes Mellitus Prescribed Insulin and/or Secretagogues: Protocol for a Prospective, 12-Wave Internet-Based Panel Survey With Email Support (the iNPHORM [Investigating Novel Predictions of Hypoglycemia Occurrence Using Real-world Models] Study)
Source: JMIR Res Protoc. 2022 Feb 11;11(2):e33726. doi: 10.2196/33726 (PMC8881777; doi:10.2196/33726)
Supplement: Multimedia Appendix 1 [file resprot_v11i2e33726_app1.docx]

Multimedia Appendix (1): Anthropometric, demographic, situational or environmental, and lifestyle variables.

|  | | | | | | | |
| --- | --- | --- | --- | --- | --- | --- | --- |
| Prognostic variable | | Questionnaire | Recall time frame | Response type | Measurement unit(s)/ Response categories^a^ | Data type | |
| Anthropometric | | | | | | | |
|  | Height | Baseline | Current | Fill-in response | Feet and inches | Continuous | |
|  | Weight | Baseline | Current | Fill-in response | Pounds | Continuous | |
|  | Body mass index | Baseline | Current | Calculated | kg/m^2^ | Continuous | |
| Demographic | | | | | | | |
|  | Living in the US | Screener and all waves | Current | Single response | Yes; No; Don’t know | Categorical | |
|  | US residence | Screener | Current | Drop-down response | State/Territory | Categorical | |
|  | Stationed at military base | Screener | Current | Single response | Yes; No |  | |
|  | Age | Screener | Current | Calculated | Years | Continuous | |
|  | Sex | Screener | Assigned at birth | Single response | Male; Female; Other | Categorical | |
|  | Gender | Screener | Current | Single response | Male; Female; Identify in another way; Prefer not to disclose | Categorical | |
|  | Race | Baseline | Current | Multi response | White; Black or African American; Asian; Hispanic, Latino/a, or Spanish origin; Native Hawaiian or other Pacific Islander; American Indian or Alaska Native; Other  Additional options if selected ‘Asian’ *or* ‘Native Hawaiian or other Pacific Islander’ | Categorical | |
|  | Ethnicity | Baseline | Current | Single response | Mexican, Mexican American, Chicano; Puerto Rican; Cuban; Another Hispanic, Latino/a, or Spanish origin (for example: Salvadoran, Dominican, Colombian, Guatemalan, Spaniard, Ecuadorian, etc.); Not of Hispanic, Latino/a, or Spanish origin | Categorical | |
|  | Marital Status | Baseline | Current | Single response | Married; Divorced; Widowed; Domestic partnership; Separated; Never married | Categorical | |
|  | Highest level of education | Baseline | Current | Single response | No schooling completed; Grades 1 through 8; Grades 9 through 12, no diploma; Regular high school diploma or GED/alternative credential; College degree or some college; Degree beyond completing first college Bachelor’s degree  Additional options if selected ‘College degree or some college’ *or* ‘Degree beyond completing first college Bachelor’s degree’ | Categorical | |
|  | Served on active duty in the USA Armed Forces | Baseline | Lifetime | Single response | Yes; No | Categorical | |
|  | Employment status | Baseline, Waves 4, 8, and 12 | Current | Single response | Working full-time, including self-employment (25 hours per week or more); Working part-time, including self-employment (less than 25 hours per week); Temporarily laid off; Temporarily unemployed due to a health-related reason; Unemployed and looking for work; Unemployed and not looking for work; Unable to work due to disability; Going to school; Looking after house/family; Retired | Categorical | |
|  | Duration of employment status | Baseline | Past 12+ months | Single response | For less than 1 month; For 1 month but less than 3 months; For 3 months but less than 6 months; For 6 months but less than 9 months; For 9 months but less than 12 months; For 12 months or longer | Categorical | |
|  | Number of people in household | Baseline | Current | Single response | 1; 2; 3; 4; 5; 6; 7; 8; 9; 10 or more | Categorical | |
|  | Sources of income (each source must contribute $\geq$10% to total household income) | Baseline | Current | Multi response | Wages, salaries, commissions, bonuses, and tips; Income from self-employment; Dividends and interest; Worker’s compensation, including Family Medical Leave Act; Employment based retirement program; Military retirement, including VA payments; Social security or other government benefits, not for disability; Social security benefits or other government benefits, specifically for disability; Child support or alimony; Other (for example: rental income, scholarships) | Categorical | |
|  | Total household income (before taxes and deductions) | Baseline | Past 12 months | Single response | Less than $10,000; $10,000 to less than $25,000; $25,000 to less than $40,000; $40,000 to less than $55,000; $55,000 to less than $70,000; $70,000 to less than $85,000; $85,000 to less than $100,000; $100,000 to less than $115,000; $115,000 to less than $130,000; $130,000 to less than $145,000; $145,000 to less than $160,000; $160,000 to less than $175,000; $175,000 to less than $200,000; $200,000 or more | Categorical | |
|  | Change in total household income | Waves 4, 8, and 12 | Past 4 months | Single response | No longer has a household income; Household income has decreased significantly (decreased by more than half but still has some income); Household income has decreased some (decreased by less than half); Household income is the same; Household income has increased some (increased by less than half); Household income has increased significantly (increased by more than half) | Categorical | |
|  | Health insurance coverage | Baseline | Current | Single response matrix  ‘Yes’ / ‘No’ / ‘Don’t know’ response categories provided for each option | Insurance through a current or former employer or union (of participant or family member) that is not a high deductible plan  Insurance purchased directly from an insurance company that is not a high deductible plan  High deductible plan  Medicare  Medicaid, Medical Assistance, or any kind of government-assistance plan  TRICARE  Veterans Affairs  Native American Health Service  Any other type of health insurance/coverage plan | Categorical | |
|  |  | Waves 4, 8, and 12 | Current | Single response matrix  ‘Yes’ / ‘No’ / ‘Don’t know’ response categories provided for each option | Insurance through a current or former employer or union (of participant or family member) that is not a high deductible plan  Insurance purchased directly from an insurance company that is not a high deductible plan  High deductible plan  Medicare  Medicaid, Medical Assistance, or any kind of government-assistance plan  TRICARE  Veterans Affairs  Native American Health Service  Consolidated Omnibus Budget Reconciliation Act (COBRA) insurance  Any other type of health insurance/coverage plan | Categorical | |
|  | Insurance coverage (other) | Baseline | Current | Fill-in response | Free-form text | String | |
|  | Duration of insurance coverage | Baseline | Past 12 months | Single response | For less than 1 month; For 1 month but less than 3 months; For 3 months but less than 6 months; For 6 months but less than 9 months; For 9 months but less than 12 months; For 12 months | Categorical | |
|  | Care received as part of a Health Maintenance Organization (HMO) | Baseline | Current | Single response | Yes; No; Don’t know | Categorical | |
|  | Co-pay assistance | Baseline | Current | Single response | Yes; No; Don’t know | Categorical | |
|  | Healthcare affordability (cost prohibitive) | Baseline | Past 12 months | Single response matrix  ‘Yes’/ ‘No’ / ‘N/A’ response categories provided for each option | Prescription medicine(s)  A treatment, such as surgery or other procedure  A medical device or medical equipment  A medical test  An appointment with a primary care doctor  An appointment with a specialist  An appointment with a healthcare provider other than a primary care doctor or specialist  Mental healthcare or counseling  Treatment or counseling for alcohol or drug use | Categorical | |
|  | Food insecurity | Baseline | Past 12 months | Single response | Yes; No | Categorical | |
| Situational/environmental | | | | | | | |
|  | Geographic area | Baseline | Current | Single response | Urban; Suburban; Rural | Categorical | |
|  | Living arrangement | Baseline | Current | Multi response | Lives alone; Lives with a spouse or partner; Lives with minor children; Lives with other adult family members; Lives with other people; Lives with pet(s) | Categorical | |
|  | Neighbourhood characteristics | Baseline | Current | Single response matrix  ‘Poor’ / ‘Fair’ / ‘Good’ / ‘Very Good’ / ‘Excellent’ response categories provided for each option | Overall rating of neighborhood as a place to live  Availability of places to buy healthy food  Ability to get around without driving a car  Availability of recreational facilities, such as parks and playgrounds  Safety from crime and violence  Overall cost of living | 5-point Likert | |
|  | Healthcare proximity (services located close enough to home) | Baseline | Current | Single response matrix  ‘Yes’ / ‘No’ / ‘Don’t know’ response categories provided for each option | A primary care doctor  A specialist  A healthcare provider other than a primary care doctor or specialist  A pharmacy  A hospital | Categorical | |
| Lifestyle | | | | | | | |
|  | Tobacco use | Baseline | Lifetime | Single response | Never; In the past but not currently; Currently | Categorical | |
|  | Recency of tobacco use | Baseline | Past 12+ months | Single response | Within the past 12 months; 12 months ago, or longer | Categorical | |
|  | Frequency of tobacco use | Baseline | Current | Single response | Less than once a month but at least once per year; 1 to 4 times per month; 2 to 6 times per week; Everyday | Categorical | |
|  | Alcohol consumption | Baseline | Lifetime | Single response | Never; In the past but not currently; Currently | Categorical | |
|  | Recency of alcohol consumption | Baseline | Past 12+ months | Single response | Within the past 12 months; 12 months ago, or longer | Categorical | |
|  | Frequency of alcohol  consumption | Baseline | Current | Single response | Less than once a month but at least once per year; 1 to 4 times per month; 2 to 6 times per week; Everyday | Categorical | |
|  | Binge drinking behaviour | Baseline | Past 30 days | Single response | 0 times; 1 time; 2 or 3 times; 4 or 5 times; More than 5 times | Categorical | |
|  | Recreational drug use | Baseline | Lifetime | Single response | Never; In the past but not currently; Currently | Categorical | |
|  | Recency of recreational drug use | Baseline | Past 12+ months | Single response | Within the past 12 months; 12 months ago, or longer | Categorical | |
|  | Frequency of recreational drug use | Baseline | Current | Single response | Less than once a month but at least once per year; 1 to 4 times per month; 2 to 6 times per week; Everyday | Categorical | |
|  | Aerobic physical activity | Baseline | Past 12 months | Single response | Never; Less than once a month but at least once per year; 1 to 4 times per month; 2 to 6 times per week; Everyday | Categorical | |
|  | Anaerobic physical activity | Baseline | Past 12 months | Single response | Never; Less than once a month but at least once per year; 1 to 4 times per month; 2 to 6 times per week; Everyday | Categorical | |
| ^a^Response categories may differ from actual questionnaire. | | | | | | |  |
