# Supplementary material for: Predicting Real-world Hypoglycemia Risk in American Adults With Type 1 or 2 Diabetes Mellitus Prescribed Insulin and/or Secretagogues: Protocol for a Prospective, 12-Wave Internet-Based Panel Survey With Email Support (the iNPHORM [Investigating Novel Predictions of Hypoglycemia Occurrence Using Real-world Models] Study)
Source: JMIR Res Protoc. 2022 Feb 11;11(2):e33726. doi: 10.2196/33726 (PMC8881777; doi:10.2196/33726)
Supplement: Multimedia Appendix 2 [file resprot_v11i2e33726_app2.docx]

Multimedia Appendix (2): Clinical variables.

|  | | | | | | | |
| --- | --- | --- | --- | --- | --- | --- | --- |
| Prognostic variable | | Questionnaire | Recall time frame | Response type | Measurement unit(s)/  Response categories^†^ | | Data type |
| Diabetes | | | | | | | |
|  | Type of diabetes | Screener | Current | Single response | Type 1 diabetes; Type 2 diabetes; Diabetes while pregnant; Don’t know; No | | Categorical |
|  |  | Baseline (verifier) | Current | Single response | Type 1 diabetes; Type 2 diabetes | | Categorical |
|  | Duration of diabetes | Baseline | Current | Calculated | Years | | Continuous |
|  | HbA1c value | Baseline | Most recent | Single response | Less than or equal to 7%; 7.1% to 8%; 8.1% to 9%; Greater than or equal to 9.1%; Don’t know | | Categorical |
|  |  | All waves | Since last iNPHORM survey was completed | (as listed above) | (as listed above) | | (as listed above) |
|  | Time since HbA1c test  (If within past 12 months) | Baseline | Past 12 months | Single response | Less than 1 month ago; Between 1 month and 3 months ago; Between 3 months and 6 months ago; Between 6 months and 9 months ago; Between 9 months and 12 months ago | | Categorical |
|  | Time since HbA1c test   (If beyond past 12 months) | Baseline | Past 12+ months | Calculated | Years | | Continuous |
|  |  | All waves | Since last iNPHORM survey was completed | Calculated | Months | | Continuous |
|  | Diabetes-related complications | Baseline | Lifetime | Single response matrix  ‘Yes’ / ‘No’ / ‘Don’t know’ response categories provided for each option | Amputation of toes, feet, or legs  Diabetes ketoacidosis  Foot damage  Gastroparesis  Hyperosmolar hyperglycemic nonketotic coma  Nephropathy  Neuropathy  Retinopathy | | Categorical |
|  |  | All waves | Since last iNPHORM survey was completed | (as listed above) | (as listed above) | | (as listed above) |
|  | Referral to kidney specialist | Baseline | Past 12 months | Single response | Yes; No | | Categorical |
|  |  | All waves | Since last iNPHORM survey was completed | (as listed above) | (as listed above) | | (as listed above) |
|  | Dialysis | Baseline | Past 12 months | Single response | Never; In the past but not currently; Currently | | Categorical |
|  |  | All waves | Current | Single response | Yes; No | | Categorical |
|  | Indicated for dialysis | Baseline and all waves | Lifetime | Single response | Yes; No | | Categorical |
| Diabetes Medications | | | | | | | |
|  | Insulin pump use | Screener and all waves | Current | Single response | Yes; No; Don’t know | Categorical | |
|  | Insulin use by type | Screener and all waves | Current | Multi response | Insulin Glargine U300; Insulin Degludec; Basal Insulin (including intermediate and long-acting); Bolus/Prandial (a.k.a. mealtime) Insulin (including rapid- and short-acting); Premixed Insulin; Fixed-Ratio Combination Insulin; Not currently taking any of these insulins | Categorical | |
|  | Insulin dose for each insulin type/pump | Screener and all waves | Yesterday | Fill-in response | Total daily units | Discrete | |
|  | Insulin duration for each insulin type/pump | Screener | Past 12+ months | Single response | For less than 1 month; For 1 month but less than 3 months; For 3 months but less than 6 months; For 6 months but less than 9 months; For 9 months but less than 12 months; For 12 months or longer; Don’t know | Categorical | |
|  |  | All waves | Since last iNPHORM survey was completed or longer | Single response | Taking insulin type/pump last iNPHORM survey was competed; Started taking insulin type/pump since the last time iNPHORM survey completed | Categorical | |
|  | Insulin duration for each insulin type/pump  (If started taking insulin type/pump since the last time iNPHORM survey was completed) | All waves | Since last iNPHORM survey was completed | Calculated | Months | Continuous | |
|  | Duration of insulin use (in general) | Screener | Lifetime | Single response | For less than 12 months; For 12 months or longer | Categorical | |
|  | Duration of insulin use (in general)  (If used for 12 months or longer) | Baseline | Lifetime | Calculated | Years | Continuous | |
|  | Secretagogue use by type | Screener and all waves | Current | Multi response | Short-Acting Sulfonylurea; Intermediate-Acting Sulfonylurea; Long-Acting Sulfonylurea; Meglitinide; Meglitinide and Biguanide Fixed-Dose Combination OR Sulfonylurea and Biguanide Fixed-Dose Combination; Not currently taking any of these secretagogues | Categorical | |
|  | Secretagogue dose for each secretagogue type | Screener and all waves | Yesterday | Fill-in response | Total daily milligrams | Discrete | |
|  | Secretagogue duration for each secretagogue type | Screener | Past 12+ months | Single response | For less than 1 month; For 1 month but less than 3 months; For 3 months but less than 6 months; For 6 months but less than 9 months; For 9 months but less than 12 months; For 12 months or longer; Don’t know | Categorical | |
|  |  | All waves | Since last iNPHORM survey was completed or longer | Single response | Taking secretagogue type the last time iNPHORM survey was completed; Started taking secretagogue type since the last time iNPHORM survey was completed | Categorical | |
|  | Secretagogue duration for each secretagogue type  (If started taking secretagogue type since the last time iNPHORM survey was completed) | All waves | Since last iNPHORM survey was completed | Calculated | Months | Continuous | |
|  | Duration of secretagogue use (in general) | Screener | Lifetime | Single response | For less than 12 months; For 12 months or longer | Categorical | |
|  | Duration of secretagogue use (in general)  (If used for 12 months or longer) | Screener | Lifetime | Calculated | Years | Continuous | |
|  | Insulin and/or secretagogue medication adherence | Baseline | Past 12 months | Single response matrix  ‘Never’ / ‘Rarely’ / ‘Sometimes’ / ‘Often’ / ‘Always’ response categories provided for each option | Forgot to take insulin and/or secretagogue dose; cut back on insulin and/or secretagogue dose without telling healthcare provider to avoid hypoglycemia; cut back on insulin and/or secretagogue dose without telling healthcare provider to avoid side effects other than hypoglycemia; did not take insulin and/or secretagogue dose at all to avoid hypoglycemia; did not take insulin and/or secretagogue dose at all to avoid side effects other than hypoglycemia; cut back on insulin and/or secretagogue dose without telling healthcare provider because felt blood glucose was under control; did not take insulin and/or secretagogue dose at all because felt blood glucose was under control | 5-point Likert | |
|  | Diabetes medications (other than insulin and secretagogues) | Baseline and all waves | Current | Multi response | Biguanide; Alpha-Glucosidase Inhibitor; Amylin Analog; Bile Acid Sequestrant; GLP-1 Receptor Agonist; Dipeptidyl Peptidase-4 (DPP-4) Inhibitor; DPP-4 Inhibitor and Biguanide Fixed-dose Combination OR DPP-4 Inhibitor and Thiazolidinedione Fixed-dose Combination; SGLT2 Inhibitor; SGLT2 Inhibitor and Biguanide Fixed-dose Combination; SGLT2 Inhibitor and DPP-4 Inhibitor Fixed-Dose Combination; Thiazolidinedione; Thiazolidinedione and Biguanide Fixed-Dose Combination; Thiazolidinedione and Sulfonylurea Fixed-Dose Combination; Not currently taking any of these diabetes medications | Categorical | |
| Diabetes management | | | | | | | |
|  | Visits with healthcare provider - diabetes discussed | Baseline | Past 12 months | Single response | 0; 1; 2; 3; 4; 5; 6; 7; 8; 9; 10; 11; 12; More than 12 visits | Categorical | |
|  |  | All waves | Since last iNPHORM survey was completed | Single response | 0; 1; 2; 3; 4; 5; 6; More than 6 visits | Categorical | |
|  | Visits with healthcare provider - hypoglycemia discussed | Baseline | Past 12 months | Single response | Every visit; Most visits; Some visits; Only a few visits; No visits | 5-point Likert | |
|  |  | All waves | Since last iNPHORM survey was completed | (as listed above) | (as listed above) | (as listed above) | |
|  | Use of blood glucose monitoring device | Baseline | Current | Single response matrix  ‘Yes’ / ‘No’ / ‘Don’t know’ response categories provided for each option | Self-monitoring blood glucose meter  Continuous or flash glucose monitoring device | Categorical | |
|  | Use of a continuous or flash glucose monitoring device | All waves | Current | Single response | Yes; No; Don’t know | Categorical | |
|  | Frequency of blood glucose testing if using self-monitoring blood glucose meter | Baseline | Past 12 months | Single response | Three or more times a day; Twice a day; Once a day; 2 to 6 times per week; 1 to 4 times per month; Less than once per month; Did not check blood glucose levels using a self-monitoring blood glucose meter | Categorical | |
|  | Time of routine blood glucose testing if using self-monitoring blood glucose meter | Baseline | Current | Single response matrix  ‘Yes’ / ‘No’ response categories provided for each option | Upon awakening and before first meal  Around mealtimes  Right before bed  Before taking diabetes medications  At another time (Free-form-text) | Categorical/ String | |
|  | Circumstances of routine blood glucose testing if using self-monitoring blood glucose meter | Baseline | Current | Single response matrix  ‘Yes’ / ‘No’ / ‘N/A’ response categories provided for each option | During or after experiencing a *hypo*glycemia event  During or after experiencing a *hyper*glycemia event  A change in diabetes medication routine  A change in work schedule  Engaging in physical activity  Traveling when driver  Traveling when not the driver  Variation in food intake  Experiencing a short-term illness  Other (Free-form-text) | Categorical/ String | |
|  | Duration of continuous or flash glucose monitoring device use | Baseline | Past 12+ months | Single response | Less than 1 month ago; Between 1 month and 3 months ago; Between 3 months and 6 months ago; Between 6 months and 9 months ago; Between 9 months and 12 months ago; More than 12 months ago | Categorical | |
|  |  | All waves | Since last iNPHORM survey was completed or longer | Single response | Using a continuous or flash glucose monitoring device the last time I completed an iNPHORM survey; I started using a continuous or flash glucose monitoring device since the last time I completed an iNPHORM survey; Don’t know | Categorical | |
|  | Duration of continuous or flash glucose monitoring device use  (If started using a continuous or flash glucose monitoring device since the last time an iNPHORM survey was completed) | All waves | Since last iNPHORM survey was completed | Calculated | Months | Continuous | |
|  | Structured diabetes education | Baseline | Lifetime | Single response | Yes; No | Categorical | |
|  | Health literacy^b^ | Baseline | Current | Single response | Modified BRIEF: Health Literacy Screening Tool [52]  3-item survey | 5-point Likert | |
|  | Sick day plan | Baseline | Lifetime | Single response | Yes; No | Categorical | |
|  | Use sick day plan | Baseline | Pro re nata | Single response | Always; Often; Sometimes; Rarely; Never | 5-point Likert | |
| General health and care | | | | | | | |
|  | Pregnancy | Screener | Current or Past 12 months | Single response | Yes; No; Don’t know | | Categorical |
|  |  | All waves | Current | (as listed above) | (as listed above) | | (as listed above) |
|  | Enrolled in interventional clinical trial or research study | Screener and all waves | Current | Single response | Yes; No; Don’t know | | Categorical |
|  | Health-related quality of life^b^ | Baseline | Past 4 weeks | Single response | VR-12 © [50,53]  12-item survey | 3-, 5-, and 6-point Likert | |
|  | Self-rated health^b^ | Baseline | Current | Single response | Self-rated health  1-item survey [51] | 5-point Likert | |
|  | Chronic co-morbidity | Baseline | Lifetime | Single response matrix  ‘Yes’ / ‘No’ / ‘Don’t know’ response categories provided for each option | Bone, joint, or muscle problem  Cancer  Cardiovascular condition  Chronic kidney disease  Chronic liver failure or liver disease  Eating disorder  Gastrointestinal disease  HIV/AIDS  Hypertension  Mental health condition  Neurological disorder  Physical impairment  Respiratory condition  Stroke or transient ischemic attack | Categorical | |
|  | Number of prescriptions  medications for chronic co-morbidity | Baseline | Current | Fill-in response | Number of prescription medications | Discrete | |
|  | Chronic condition that impacts hypoglycemia management | Baseline | Current | Single response | Yes; No | Categorical | |
|  | Use of corticosteroids | Baseline | Current | Single response | Yes; No; Don’t know | Categorical | |
|  | Duration of corticosteroids | Baseline | Past 12+ months | Single response | For less than 1 month; For 1 month but less than 3 months; For 3 months but less than 6 months; For 6 months but less than 9 months; For 9 months but less than 12 months; For 12 months or longer | Categorical | |
|  | Use of beta-blockers | Baseline | Current | Single response | Yes; No; Don’t know | Categorical | |
|  | Duration of beta-blockers | Baseline | Past 12+ months | Single response | For less than 1 month; For 1 month but less than 3 months; For 3 months but less than 6 months; For 6 months but less than 9 months; For 9 months but less than 12 months; For 12 months or longer | Categorical | |
|  | Use of antibiotics | Baseline | Current | Single response | Yes; No; Don’t know | Categorical | |
|  | Duration of antibiotics | Baseline | Past 12+ months | Single response | For less than 1 month; For 1 month but less than 3 months; For 3 months but less than 6 months; For 6 months but less than 9 months; For 9 months but less than 12 months; For 12 months or longer | Categorical | |
|  | Use of corticosteroids, beta-blockers, antibiotics | All waves | Current | Multi response | Corticosteroids; Beta-blockers; Antibiotics | Categorical | |
|  | Duration of corticosteroids | All waves | Since last iNPHORM survey was completed or longer | Single response | Taking medication type the last time completed an iNPHORM survey; Started taking medication type since the last time completed an iNPHORM survey | Categorical | |
|  | Duration of corticosteroids  (If started corticosteroids since the last time an iNPHORM survey was completed) | All waves | Since last iNPHORM survey was completed | Calculated | Months | Continuous | |
|  | Duration of beta-blockers | All waves | Since last iNPHORM survey was completed or longer | Single response | Taking medication type the last time completed an iNPHORM survey; Started taking medication type since the last time completed an iNPHORM survey | Categorical | |
|  | Duration of beta-blockers  (If started beta-blockers since the last time an iNPHORM survey was completed) | All waves | Since last iNPHORM survey was completed | Calculated | Months | Continuous | |
|  | Duration of antibiotics | All waves | Since last iNPHORM survey was completed or longer | Single response | Taking medication type the last time completed an iNPHORM survey; Started taking medication type since the last time completed an iNPHORM survey | Categorical | |
|  | Duration of antibiotics  (If started antibiotics since the last time an iNPHORM survey was completed) | All waves | Since last iNPHORM survey was completed | Calculated | Months | Continuous | |
| HbA1c, Hemoglobin A1c  ^a^Response categories may differ from actual questionnaires  ^b^Patient-reported outcome | | | | | | | |
