# Supplementary material for: Predicting Real-world Hypoglycemia Risk in American Adults With Type 1 or 2 Diabetes Mellitus Prescribed Insulin and/or Secretagogues: Protocol for a Prospective, 12-Wave Internet-Based Panel Survey With Email Support (the iNPHORM [Investigating Novel Predictions of Hypoglycemia Occurrence Using Real-world Models] Study)
Source: JMIR Res Protoc. 2022 Feb 11;11(2):e33726. doi: 10.2196/33726 (PMC8881777; doi:10.2196/33726)
Supplement: Multimedia Appendix 3 [file resprot_v11i2e33726_app3.docx]

Multimedia Appendix (3): COVID-19–related variables.

|  | |  |  |  |  |  | |
| --- | --- | --- | --- | --- | --- | --- | --- |
| Prognostic variable | | Questionnaire^a^ | Recall time frame | Response type | Measurement unit(s)/  Response categories^b^ | Data type | |
| COVID-19 infection status | | | | | | |  |
|  | COVID-19 symptoms and epidemiologic exposure | Sub-panel A:  Waves 2-12  Sub-panel B:  Waves 1-12 | Since last iNPHORM survey was completed | Single response matrix  ‘Yes’ / ‘No’ response categories provided for each option | Cough, difficulty breathing, and/or fever (over 100 degrees Fahrenheit)  Symptoms typical of COVID-19 such as sore throat, headache, tiredness, or muscle aches and pains  Had close contact with someone who has been tested and is confirmed to have COVID-19  Had close contact with someone who has been tested for COVID-19 and does not know the results of the test yet  Had close contact with someone who is ill with cough and/or fever that travelled outside of the US prior to feeling ill  Travelled outside of the US | Categorical | |
|  | COVID-19 diagnosis | Sub-panel A:  Waves 2-12  Sub-panel B:  Waves 1-12 | Since last iNPHORM survey was completed | Single response | Confirmed by a test from a healthcare professional; Suspected but not confirmed by a test from a healthcare professional; Neither confirmed nor suspected | Categorical | |
| Community containment because of COVID-19 infection/situation | | | | | | |  |
|  | Social/physical distancing or shelter-in-place | Sub-panel A:  Waves 2-12  Sub-panel B:  Waves 1-12 | Since last iNPHORM survey was completed | Single response | Always; Often; Sometimes; Rarely; Never | 5-point Likert | |
|  | Self-quarantine or self-isolation | Sub-panel A:  Waves 2-12  Sub-panel B:  Waves 1-12 | Since last iNPHORM survey was completed | Single response | Currently in self-quarantine/self-isolation; Previously in self-quarantine/self-isolation; Not been in self-quarantine/self-isolation | Categorical | |
| Impact of COVID-19 situation on financial well-being | | | | | | |  |
|  | Impact of COVID-19 situation on employment status, total household income, or health insurance income | Waves 4, 8, and 12 | Current/Past 4 months | Single response matrix  ‘Yes’ / ‘No’ response categories provided for each option | Current employment status has been impacted  Total household income within the past 4 months has been impacted  Current health insurance coverage has been impacted | Categorical | |
|  | Impact of COVID-19 situation on economic well-being in general | Waves 4, 8, and 12 | Past 4 months | Fill-in response | Free-form-text | String | |
| Impact of COVID-19 situation on diabetes management and outcomes | | | | | | |  |
|  | Impact of COVID-19 situation on various aspects glycemic management | Sub-panel A:  Waves 2-12  Sub-panel B:  Waves 1-12 | Since last iNPHORM survey was completed | Single response matrix  ‘...has been much harder ‘/ ‘...has been somewhat harder’ / ‘...has not been impacted’ / ‘... has been somewhat easier’ / ‘...has been much easier’ response categories provided for each option | Affording rent and other living expenses...  Affording diabetes medication(s)...  Affording test strips and/or sensors...  Retrieving diabetes medication(s) from the pharmacy...  Ensuring enough food to avoid hypoglycemia...  Testing/monitoring blood glucose...  Staying as physically active as usual...  Consulting with healthcare provider(s) about diabetes...  Remembering to take diabetes medication(s) as prescribed...  Monitoring risk of hypoglycemia regularly...  Having enough social support to help manage hypoglycemia...  Feeling in control of hypoglycemia... | 5-point Likert | |
|  | Impact of COVID-19 situation on drug rationing to preserve medical supplies | Sub-panel A:  Waves 2-12  Sub-panel B:  Waves 1-12 | Since last iNPHORM survey was completed | Single response | Yes; No | Categorical | |
|  | Impact of COVID-19 situation on drug rationing to avoid hypoglycemia | Sub-panel A:  Waves 2-12  Sub-panel B:  Waves 1-12 | Since last iNPHORM survey was completed | Single response | Yes; No | Categorical | |
|  | Impact of COVID-19 situation on hypoglycemia frequency | Sub-panel A:  Waves 2-12  Sub-panel B:  Waves 1-12 | Since last iNPHORM survey was completed | Single response | Experienced far more hypoglycemia events; Experienced somewhat more hypoglycemia events; Number of hypoglycemia events has not been impacted; Experienced somewhat fewer hypoglycemia events; Experienced far fewer hypoglycemia events | 5-point Likert | |
|  | Impact of COVID-19 situation on diabetes in general | Sub-panel A:  Waves 2-12  Sub-panel B:  Waves 1-12 | Since last iNPHORM survey was completed | Fill-in response | Free-form-text | String | |
| ^a^The COVID-19 sub-questionnaire was administered first to Sub-panel A at Wave 2 (April 2020)  ^b^Response categories may differ from actual questionnaire | | | | | | |  |
