# Supplementary material for: Predicting Real-world Hypoglycemia Risk in American Adults With Type 1 or 2 Diabetes Mellitus Prescribed Insulin and/or Secretagogues: Protocol for a Prospective, 12-Wave Internet-Based Panel Survey With Email Support (the iNPHORM [Investigating Novel Predictions of Hypoglycemia Occurrence Using Real-world Models] Study)
Source: JMIR Res Protoc. 2022 Feb 11;11(2):e33726. doi: 10.2196/33726 (PMC8881777; doi:10.2196/33726)
Supplement: Multimedia Appendix 4 [file resprot_v11i2e33726_app4.docx]

Multimedia Appendix (4): Hypoglycemia-related variables.

|  | | | | | | |
| --- | --- | --- | --- | --- | --- | --- |
| Prognostic variable | | Questionnaire | Recall time frame | Response type | Measurement unit(s)/  Response categories^a^ | Data type |
| Hypoglycemia symptoms | | | | | | |
|  | Symptomology | Baseline | Current | Multi response | Aura; Mood swings; Fatigue; Shakiness; Dizziness; Headache; Sudden hunger; Food cravings; Cravings for sweets; Mental confusion; Depression; Nervousness; Heart palpitations; Blurred vision; Phobias; Cold hands or feet; Outbursts of temper; Crying; Insomnia; Loss of consciousness; Other; None; Don’t know | Categorical |
|  |  | All waves | Since last iNPHORM survey was completed | (as listed above) | (as listed above) | (as listed above) |
|  | Impaired awareness of hypoglycemia^b^ | Baseline | Current | Single response | Modified Clarke method  8-item survey [61] | Categorical |
|  |  | All waves | Current/Since last iNPHORM survey was completed | (as listed above) | (as listed above) | (as listed above) |
|  |  | Wave 6 | Current | Single response | Gold method  1-item survey [62] | Categorical |
| Non-severe hypoglycemia: nature, frequency, and treatment | | | | | | |
|  | Total frequency of non-severe hypoglycemia  (Question asked separately for daytime non-severe and nocturnal non-severe hypoglycemia) | Baseline | Past 30 days | Fill-in response | Frequency | Discrete |
|  |  | All waves | Past 30 days or since last iNPHORM survey was completed | (as listed above) | (as listed above) | (as listed above) |
|  | Identification of non-severe hypoglycemia  (Question asked separately for daytime non-severe and nocturnal non-severe hypoglycemia) | Baseline | Past 30 days | Fill-in single response matrix | Frequencies reported for events identified by:  Symptoms without a measured blood glucose value  Measured blood glucose value without symptoms  Both symptoms and a measured blood glucose value  Don’t know | Discrete |
|  |  | All waves | Past 30 days or since last iNPHORM survey was completed | (as listed above) | (as listed above) | (as listed above) |
|  | Cause of non-severe hypoglycemia  (Question asked separately for daytime non-severe and nocturnal non-severe hypoglycemia) | Baseline | Past 30 days | Multi response | Variation in food intake; Exercise; Incorrect insulin and/or secretagogue use; Other (Free-form-text option); Don’t know | Categorical /String |
|  |  | All waves | Past 30 days or since last iNPHORM survey was completed | (as listed above) | (as listed above) | (as listed above) |
|  | Methods used to treat non-severe hypoglycemia  (Question asked separately for daytime non-severe and nocturnal non-severe hypoglycemia) | Baseline | Past 30 days | Multi response | Juice or a soft drink; Glucose or sucrose taken orally; Candies; A snack; A meal; Other (Free-form-text option); None (Recovered without treatment); Don’t know | Categorical /String |
|  |  | All waves | Past 30 days or since last iNPHORM survey was completed | (as listed above) | (as listed above) | (as listed above) |
|  | Use of second treatment because unable to treat non-severe hypoglycemia effectively  (Question asked separately for daytime non-severe and nocturnal non-severe hypoglycemia) | Baseline | Past 30 days | Single response | Never; Rarely; Sometimes; Often; Always | 5-point Likert |
|  |  | All waves | Past 30 days or since last iNPHORM survey was completed | (as listed above) | (as listed above) | (as listed above) |
|  | Blood glucose level when non-severe hypoglycemia is experienced | Baseline | Current | Fill-in response | mg/dl or less | Continuous |
|  |  | All waves | Past 30 days or since last iNPHORM survey was completed | (as listed above) | (as listed above) | (as listed above) |
| Severe hypoglycemia: nature, frequency, and treatment | | | | | | |
|  | Total frequency of severe hypoglycemia  (Question asked separately for daytime severe and nocturnal severe hypoglycemia) | Baseline  All waves | Past 12 months  Since last iNPHORM survey was completed | Fill-in response  (as listed above) | Frequency  (as listed above) | Discrete  (as listed above) |
|  | Treatment location of severe hypoglycemia  (Question asked separately for daytime severe and nocturnal severe hypoglycemia) | Baseline | Past 12 months | Fill-in single response matrix | Frequencies reported for each of the following:  Treated outside of a hospital by a person who is not a healthcare provider  Treated outside of a hospital by a paramedic, doctor, or other healthcare provider  Treated in the emergency department of a hospital. No hospital admission.  Treated in the emergency department of a hospital. Hospital admission  Recovered spontaneously  Treated in another way  Don’t know | Discrete |
|  |  | All waves | Since last iNPHORM survey was completed | (as listed above) | (as listed above) | (as listed above) |
|  | Frequency of unconsciousness due to severe hypoglycemia  (Question asked separately for daytime severe and nocturnal severe hypoglycemia) | Baseline | Past 12 months | Fill-in response | Frequency | Discrete |
|  |  | All waves | Since last iNPHORM survey was completed | (as listed above) | (as listed above) | (as listed above) |
|  | Cause of severe hypoglycemia  (Question asked separately for daytime severe and nocturnal severe hypoglycemia) | Baseline | Past 12 months | Multi response | Variation in food intake (for example: missed or delayed meals, less carbohydrate intake, etc.); Exercise; Incorrect insulin and/or secretagogue use; Other (Free-form-text option); Don’t know | Categorical /String |
|  |  | All waves | Since last iNPHORM survey was completed | (as listed above) | (as listed above) | (as listed above) |
|  | Treatment of severe hypoglycemia  (Question asked separately for daytime severe and nocturnal severe hypoglycemia) | Baseline | Past 12 months | Multi response | Glucagon injection; Glucagon nasal spray; Glucose injection; Glucose or sucrose taken orally; Other (Free-form-text option); Don’t know | Categorical /String |
|  |  | All waves | Since last iNPHORM survey was completed | (as listed above) | (as listed above) | (as listed above) |
| Hypoglycemia management | | | | | | |
|  | Self-management behaviour | Baseline | Current | Single response matrix  ‘Never’ / ‘Rarely’ / ‘Sometimes’ / ‘Often’ /  ‘Always’ response categories provided for each option | Adjust diabetes medications that have been recommended by a healthcare provider to avoid hypoglycemia.  Adjust diabetes medications that have *not* been recommended by a healthcare provider to avoid hypoglycemia.  Monitor risk of hypoglycemia regularly.  Take measures to avoid hypoglycemia when exercising.  Monitor food/carbohydrate intake to avoid hypoglycemia.  Treat hypoglycemia with the appropriate amount of food/carbohydrates.  Check blood glucose level 15 minutes after treating a hypoglycemia event to ensure no longer low.  Keep glucagon on-hand for emergency. | 5-point Likert |
|  | Hypoglycemia detection because of continuous or flash glucose monitoring device | Baseline | Current | Single response | Detect a higher number of hypoglycemia events while using a continuous or flash glucose monitoring device; Detect the same number of hypoglycemia events while using a continuous or flash glucose monitoring device; Detect a lower number of hypoglycemia events while using a continuous or flash glucose monitoring device; N/A | 3-point Likert |
|  |  | All waves  (If started using continuous or flash glucose monitoring device since the last iNPHORM survey was completed) | Since last iNPHORM survey was completed | (as listed above) | (as listed above) | (as listed above) |
|  | Social support to help manage hypoglycemia | Baseline | Current | Single response | Yes; No | Categorical |
|  | Describe relationship type with social support | Baseline | Current | Fill-in response | Free-form-text | String |
|  | Social support lives with respondent | Baseline | Current | Single response | Yes; No | Categorical |
|  | Social support’s level of commitment | Baseline | Current | Single response | Far too much; Too much; The right amount; Too little; Far too little | 5-point Likert |
|  | Social support knows how to administer glucagon | Baseline | Current | Single response | Yes; No | Categorical |
|  | Fear of hypoglycemia^b^ | Wave 6 | Past 6 months | Single response | Hypoglycemia Fear Survey II (HFS-II) [63]  33-item survey | 5-point Likert |
|  | Modifiable factors of hypoglycemia self-management behaviour^b^ | Wave 6 | Current | Single response | InHypo-DM Person with Diabetes Questionnaire [60]  61-item survey | 5-point Likert |
| ^a^ Response categories may differ from actual questionnaire  ^b^ Patient-reported outcome | | | | | | |
