# Supplementary material for: Predicting Real-world Hypoglycemia Risk in American Adults With Type 1 or 2 Diabetes Mellitus Prescribed Insulin and/or Secretagogues: Protocol for a Prospective, 12-Wave Internet-Based Panel Survey With Email Support (the iNPHORM [Investigating Novel Predictions of Hypoglycemia Occurrence Using Real-world Models] Study)
Source: JMIR Res Protoc. 2022 Feb 11;11(2):e33726. doi: 10.2196/33726 (PMC8881777; doi:10.2196/33726)
Supplement: Multimedia Appendix 5 [file resprot_v11i2e33726_app5.docx]

| Study  Title | Investigating Novel Predictions of Hypoglycemia Occurrence using Real-World Models -  the **iNPHORM**Study |
| --- | --- |
| Principal Investigator | Dr. Stewart Harris, Professor  Department of Family Medicine at Western University, London, Ontario, Canada  519-661-2111 extension 22057 |
| Funder | Sanofi Canada |
| Conflict of Interest | Dr. Harris receives consulting fees from Sanofi for participating in advisory boards, symposiums, and clinical trials. |

 Multimedia Appendix (5): Letter of information and consent emailed to prospective participants of the iNPHORM (Investigating Novel Predictions of Hypoglycemia Occurrence Using Real-world Models) pilot study.

Please save/print this letter for your reference before moving to the consent section

Introduction:

Low blood sugar (also known as hypoglycemia) is the most common adverse event of insulin and secretagogue use in people with diabetes. It can result in distressing and sometimes severe physical and social consequences. A better understanding of who is at greatest risk of diabetes-related hypoglycemia, and why, is needed.

The purpose of the iNPHORM study is 1) to understand how often people with diabetes have hypoglycemia; and 2) to build a tool that healthcare providers can use to predict the chance that a given person with diabetes will have hypoglycemia in the future.

The purpose of the **testing phase** of the iNPHORM study is to evaluate the surveys that will be used.

You are invited to participate in the **testing phase** of the iNPHORM study because you are: 1) an existing member of a survey panel consenting to be contacted for research surveys; and 2) have been prescribed insulin or secretagogues to treat your diabetes.  The testing phase will take 2 weeks to complete.

What will be requested of me as a participant?

If you choose to participate, your role will be to test and provide your feedback on surveys that have been developed for the iNPHORM study at an online/telephone testing session where an interviewer will ask you questions about the length, clarity, and overall design of the surveys.

You will be asked to complete and provide feedback on:

- A survey that will be used to screen people to determine whether they are qualified to participate.  This is the same qualification screening survey that you just completed.
- A survey that will be used to gather initial information on participants. The survey includes questions about yourself, your general health, diabetes health, medication use, and experience(s) with hypoglycemia.

**In addition,**you might be asked to review or complete and provide feedback on:

- A short survey that will be sent out on a monthly basis to gather updated health information. The survey includes questions about your health, diabetes, medication use, and experience(s) with hypoglycemia.
- An email that will notify the iNPHORM participant of the next survey

OR

- A survey that will be used to gather information on (1) how hypoglycemia affects emotions; (2) thoughts, feelings, beliefs, and actions around hypoglycemia management; and (3) updated health information including questions health, diabetes, medication use, experience(s) with hypoglycemia.
- An email that will notify the iNPHORM participant of the next survey

What will happen during this study?

- If, after reading this letter, you consent to participate you will receive a confirmation message. There will be only three people with diabetes involved in this testing phase therefore once three individuals have agreed to participate, no more participants will be enrolled into this study. It is possible that you may submit your information but not be enrolled into this study. If this is the case you will receive a second email informing you that enrollment is full.
- Once enrolled, you will receive an email confirming the date of your testing session and how to access the session. You will also receive reminders before the session.
- The testing session will be hosted over an online screen-sharing platform and by telephone by a staff person from Ipsos Healthcare who will observe you completing the surveys and obtain your feedback on them.  The testing session will take 60- 90 minutes.
- Your responses to the original qualification screening survey will be used as part of this study.
- To thank you for your time and effort in completing the testing session, you’ll receive a $300 honorarium credited to your OpinionSite portal.

What will be the risks and benefits of participating?

You may not directly benefit from participating in this study but your responses will help improve the design of the surveys for future use. There are no major risks associated with participating in this study however, you may be reminded of stressful circumstances when describing your experience(s) with hypoglycemia in these surveys. There is a very minimal risk of privacy breach of the Ipsos servers for the personal identifying data (your phone number, full birthdate) collected as part of the study.

What are my rights as a participant?

Your participation is completely voluntary. You may refuse to answer any survey questions you do not want to answer, or any interview questions by saying “pass”.

You do not waive any legal rights by consenting to this study. You have the right withdraw from the study at any time by:

- Unsubscribing - clicking on the unsubscribe link at the bottom of any email communication, or
- Emailing the moderator at Daniel.Buchenberger@ipsos.com, or
- Telling your interviewer during the interview

By withdrawing, your survey responses and interview responses collected before you leave the study will still be used to improve the surveys. No new information will be collected without your permission.

How will my confidentiality be maintained?

All of your responses will be kept strictly confidential.  You will be given a unique participant ID number when you enroll in this study. Your survey responses and interview responses will only be linked to your participant ID number.

OpinionSite will send your responses to the qualification screening survey and your personal identifiers (phone number, full birthdate) to Ipsos so you can be personally addressed in the testing session. After the testing session the data gathered will be de-identified, meaning Ipsos will remove the personal identifying data prior to sending it to Dr. Stewart Harris at Western University. Only members of Dr. Harris’s research team will have access to the data. As your data is de-identified, you will not be named in any reports, publications, or presentations.

OpinionSite will archive your responses to the qualification screening survey for 5 years. Ipsos will archive your survey responses and your personal identifying data for 7 years. The research team at Western University will keep your de-identified survey responses for 7 years.

Who do I contact with my questions?

If you have any questions about the OpinionSite survey platform or how the study operates from a technical standpoint, please email **qual@opinionsite.com**.

If you have any questions or concerns about the content of the research study, please contact Susan Webster-Bogaert from Dr. Stewart Harris’s Research Team (telephone 1-855-858-6872 email: mwebster@uwo.ca).

If you have any questions about your rights as a participant or the conduct of this study, you may contact the Office of Human Research Ethics at Western University (toll-free telephone #: 1-844-720-9816 or email: ethics@uwo.ca). The Office of Human Research Ethics is a group of people who oversee the ethical conduct of research studies. They are not part of the research team or OpinionSite or Ipsos. Everything that you discuss will be kept confidential.

Please save/print this letter for your reference before moving to the consent section.

You may also email qual@opinionsite.com for a copy of this letter.

[After reading the Letter of Information, the participants will click on a “Next” button that will direct them to a separate webpage to obtain consent]

I have reviewed the Letter of Information and understand my role. I know that I may leave the study at any time.

- I agree to participate
- I do not agree to participate
