# Supplementary material for: Predicting Real-world Hypoglycemia Risk in American Adults With Type 1 or 2 Diabetes Mellitus Prescribed Insulin and/or Secretagogues: Protocol for a Prospective, 12-Wave Internet-Based Panel Survey With Email Support (the iNPHORM [Investigating Novel Predictions of Hypoglycemia Occurrence Using Real-world Models] Study)
Source: JMIR Res Protoc. 2022 Feb 11;11(2):e33726. doi: 10.2196/33726 (PMC8881777; doi:10.2196/33726)
Supplement: Multimedia Appendix 6 [file resprot_v11i2e33726_app6.docx]

### Multimedia Appendix (6): Letter of information and consent emailed to prospective participants of the iNPHORM (Investigating Novel Predictions of Hypoglycemia Occurrence Using Real-world Models) longitudinal study.

| Study  Title | Investigating Novel Predictions of Hypoglycemia Occurrence using Real-World Models -  the **iNPHORM**Study |
| --- | --- |
| Principal Investigator | Dr. Stewart Harris, Professor  Department of Family Medicine at Western University, London, Ontario, Canada  519-661-2111 extension 22057 |
| Funder | Sanofi Canada |
| Conflict of Interest | Dr. Harris receives consulting fees from Sanofi for participating in advisory boards, symposiums, and clinical trials. |

Please save or print this letter for your reference before moving to the consent section

Introduction:

Low blood sugar (also known as hypoglycemia) is the most common adverse event of insulin and secretagogue use in people with diabetes. It can result in distressing and sometimes severe physical and social consequences. A better understanding of who is at greatest risk of diabetes-related hypoglycemia, and why, is needed.

The purpose of the iNPHORM study is: 1) to understand how often people with diabetes have hypoglycemia; and 2) to build a tool that healthcare providers can use to predict the chance that a given person with diabetes will have hypoglycemia in the future.

You are invited to participate in the iNPHORM study because you are: 1) an existing member of a survey panel consenting to be contacted for research surveys; and 2) have been prescribed insulin or secretagogues to treat your diabetes. There will be at least 1250 people with diabetes from all over the United States of America involved in this 16-month survey study.

What will be requested of me as a participant?

If you choose to participate, you will be involved for 12 months and be asked to:

1. Complete an initial survey to share some information about yourself, your general health, diabetes health, medication use, and experience(s) with hypoglycemia. This survey will take approximately 30 minutes to complete.
2. Join the iNPHORM Diabetes Community, so you can be contacted each month for the next 12 months to complete surveys for the iNPHORM study. This community will not put you in contact with other participants.
3. Complete a series of short, 10 minute, monthly surveys sharing updates about your health and experience(s) with hypoglycemia.
4. Complete a longer 30 minute survey at 6 months sharing
5. how hypoglycemia affects your emotions
6. your thoughts, feelings, beliefs, and actions around hypoglycemia management
7. an update of your health and experience(s) with hypoglycemia.

What will happen during this study?

- If, after reading this letter, you consent to participate you will then start the process to join the iNPHORM Diabetes Community by reading and agreeing to abide by Ipsos’ community Terms and Conditions and Privacy Policy.
- Next you will be directed to the initial survey. You will be able to save your place while filling out the survey.  To do this you will click the *Need a break?* checkbox and then you will be asked to enter your email address. You will be emailed a link to your saved survey. Note however that the number of participants is limited to 1250 and you will be only enrolled in the study after the initial survey is complete and you have joined the iNPHORM Diabetes Community.
- Once you submit the initial survey you will be directed to enter and confirm your email address to continue the process of joining the iNPHORM Diabetes Community.
- You will be directed to a registration webpage where you will be asked to provide some information. Please only provide: 1) a username, 2) a password, 3) your time zone, and 4) your first name, so emails can be addressed to you personally. Please leave all other boxes blank.
- Each month, you will receive an email to notify you that the survey will be sent in a week. Seven days later, you will receive an email containing a link to the survey. The link will be active for 7 days. You will receive two reminder emails if you have not completed the survey.
- For the **first** monthly survey, if you do not complete the survey within the 7 days, **you will be withdrawn from the study.**
- For all other months if you do not complete a survey within the 7 days, you will still have the opportunity to fill out a survey the next month.

Note if you consent to participate, your responses to the qualification screening survey will also be used as part of this study.

Are participants paid to be in this study?

Participants are not paid to be in the study however honorarium gift-cards are offered to thank you for your time and effort.

- For completing the initial survey, you will receive the honorarium as shown in the email by the site that invited you to participate.
- For completing each monthly survey, you will receive a $10 honorarium giftcard. At the end of the year, if you complete 8-11 monthly surveys, you’ll receive an additional $30 honorarium giftcard; if you complete all 12 monthly surveys, you'll receive a $75 honorarium giftcard.
- In addition, there will be two draws:
- For completing the 6^th^ monthly survey, you will be entered into a draw where three winners will be randomly selected to receive a $500 honorarium giftcard each.
- For completing the 12^th^ (also the last) monthly survey, you will be entered into a draw where three winners will be randomly selected to receive a $1000 honorarium giftcard each.

What will be the risks and benefits of participating?

You may not directly benefit from participating in this study but your responses may guide improvements to future diabetes management strategies.

There are no major risks associated with participating in this study. However, you may be reminded of stressful circumstances when describing your experience(s) with hypoglycemia in the surveys. There is a very minimal risk of privacy breach of the Ipsos servers for any personal identifying data collected in this study as part of registration or completing the surveys (name, date of birth, email address).

What are my rights as a participant?

Your participation is voluntary. You may decide not to be in this study, or to be in the study now and then change your mind later. You may refuse to answer any question you do not want to answer.

You do not waive any legal rights by consenting to this study. You have the right to withdraw from the study at any time by:

- Unsubscribing - clicking on the unsubscribe link at the bottom of any email communication, or
- Emailing the moderator at support@ipsossrs.com

If you decide to withdraw or if you are withdrawn because you did not complete the first monthly survey, you will be removed from the iNPHORM Diabetes Community but all past survey responses including the qualification screening survey will still be used as part of this study.

How will my confidentiality be maintained?

All of your responses will be kept strictly confidential.  You will be given a unique participant ID number when you join the iNPHORM Diabetes Community. Your survey responses will only be linked to your participant ID number.

Your survey data will be de-identified, meaning Ipsos will remove any personal identifying data collected in this study as part of registration or completing the surveys (name, email address, and full birthdate). This survey data will be sent to Dr. Stewart Harris at Western University. Only members of Dr. Harris’s research team will have access to the data. As the data sent to Western University are de-identified, you will not be named in any reports, publications, or presentations that may result from this research study.

Dr. Harris is required to share information on severe hypoglycemia events and other significant health events with drug manufacturers. For respondents prescribed *Toujeo® SoloSTAR®* or *Toujeo® Max SoloStar®*anonymized data will be sent to Sanofi and for those prescribed*Tresiba® FlexTouch® U-100* or *Tresiba® FlexTouch® U-* *200* anonymized data will be sent to Novo Nordisk.

Ipsos will store your survey responses for 7 years. The research team at Western University will keep the de-identified survey data for 7 years.

Who do I contact with my questions?

If you have any questions about the Ipsos survey platform or how the study operates from a technical standpoint, please email support@ipsossrs.com.

If you have any questions or concerns about the content of the research study, please contact Susan Webster-Bogaert from Dr. Stewart Harris’s Research Team (telephone 1-855-858-6872 email: mwebster@uwo.ca).

If you have any questions about your rights as a participant or the conduct of this study, you may contact the Office of Human Research Ethics at Western University (toll-free telephone #: 1-844-720-9816 or email: ethics@uwo.ca). The Office of Human Research Ethics is a group of people who oversee the ethical conduct of research studies. They are not part of the research team or Ipsos. Everything that you discuss will be kept confidential.

Please save or print this letter for your reference before moving to the consent section.

You may also email support@ipsossrs.com for a copy of this letter.

[After reading the Letter of Information, the participants will click on a “Next” button that will direct them to a separate webpage with the following consent question.] 

I have reviewed the Letter of Information and understand that the study includes completing an initial survey plus 12 surveys over 12 months. I know that I may leave the study at any time.

- I agree to participate
- I do not agree to participate
