# Supplementary material for: Predicting Real-world Hypoglycemia Risk in American Adults With Type 1 or 2 Diabetes Mellitus Prescribed Insulin and/or Secretagogues: Protocol for a Prospective, 12-Wave Internet-Based Panel Survey With Email Support (the iNPHORM [Investigating Novel Predictions of Hypoglycemia Occurrence Using Real-world Models] Study)
Source: JMIR Res Protoc. 2022 Feb 11;11(2):e33726. doi: 10.2196/33726 (PMC8881777; doi:10.2196/33726)
Supplement: Multimedia Appendix 7 [file resprot_v11i2e33726_app7.docx]

Multimedia Appendix (7): Calculation of average total completion rate.

Average total completion rate was calculated by comparing the observed number of completed follow-up questionnaires to the maximum expected number (i.e., completion of all follow-up questionnaires by all individuals):

$$= \left( \frac{\text{Actual number of follow-up questionnaires completed}}{\text{Expec}\text{ted number of follow-up questionnaires completed under complete follow-up}} \right)\text{*100\%}$$

$$= \left[ \frac{\text{10,470 follow up questionnaires}}{\text{(1,206 individuals*12 months)}} \right]\text{*100\%}$$

$$= \left( \frac{\text{10,470}}{\text{14,472}} \right)*100\%$$

$$= \text{72.4\%}$$
