# Supplementary material for: Predicting Real-world Hypoglycemia Risk in American Adults With Type 1 or 2 Diabetes Mellitus Prescribed Insulin and/or Secretagogues: Protocol for a Prospective, 12-Wave Internet-Based Panel Survey With Email Support (the iNPHORM [Investigating Novel Predictions of Hypoglycemia Occurrence Using Real-world Models] Study)
Source: JMIR Res Protoc. 2022 Feb 11;11(2):e33726. doi: 10.2196/33726 (PMC8881777; doi:10.2196/33726)
Supplement: Multimedia Appendix 8 [file resprot_v11i2e33726_app8.docx]

Multimedia Appendix (8): Calculation of average total completion rate against estimated required sample size (N=521).

Recall that iNPHORM follow-up questionnaires assessed individuals’ number of severe hypoglycemia events since their last completed questionnaire and, as such, complete information on severe hypoglycemia events is available for all individuals up until their last completed questionnaire (after this point, they were considered “right-censored”). Participants were considered under observation for severe hypoglycemia events from baseline until their last completed questionnaire. Therefore, our completion rate as compared to our estimated required sample size (N=521) was calculated as follows:

$$= \left( \frac{\text{Observed sum of person-months for severe hypoglycemia}}{\text{Expected sum of person-months for severe hypoglycemia}\text{ }\text{under complete follow-up for estimated required sample size}} \right)*100\%$$

$$\text{= }\left[ \frac{\text{11,192 person-months}}{\left( \text{521 individuals*12 months} \right)\text{ }} \right]\text{*100\%}$$

$$\text{=} \left( \frac{\text{11,192}}{\text{6,252}} \right)\text{*100\%}$$

$$\text{= 179.0\%}$$
